# Development of the European Laparoscopic Intermediate Urological Skills LUSs2 Curriculum: A Delphi Consensus from the European School of Urology

**DOI:** 10.1016/j.euros.2024.08.023

**Published:** 2024-09-13

**Authors:** Diego M. Carrion, Loic Baekelandt, Moises Rodriguez Socarras, Willem M. Brinkman, Tiago Ribeiro de Oliveira, Giovannalberto Pini, Anna H. de Vries, Cristina E. Bujoreanu, Tomasso Silvestri, Andreas Skolarikos, Bogdan Petrut, Domenico Veneziano, Francesco Greco, Mario Alvarez-Maestro, Rafael Sanchez-Salas, Rafael Rocha Tourinho-Barbosa, Evangelos Liatsikos, Bhaskar Somani, Juan Gomez Rivas, Paticia J. Zondervan

**Affiliations:** a Department of Urology, Torrejon University Hospital, Madrid, Spain; b Universidad Francisco de Vitoria, Madrid, Spain; c Department of Urology, University Hospitals Leuven, Leuven, Belgium; d Instituto de Cirugía Urológica Avanzada (ICUA), Clínica CEMTRO, Madrid, Spain; e Department of Oncological Urology, University Medical Centrum Utrecht, Utrecht, The Netherlands; f Department of Urology, Armed Forces Hospital, Lisbon, Portugal; g Department of Urology, Ospedale San Raffaele-Turro, Milan, Italy; h Department of Urology, Diakonessenhuis, Utrecht, The Netherlands; i Department of Urology, Medicover Hospital, Cluj-Napoca, Romania; j Department of Urology, Trieste University, Trieste, Italy; k Second Department of Urology, Sismanoglio Hospital, Athens Medical School, Athens, Greece; l Department of Urology, Institutul Oncologic Cluj Napoca, District Cluij, Romania; m The Smith Institute for Urology, Northwell Health, New York, NY, USA; n Department of Urology, Centro Salute Uomo, Bergamo, Italy; o Department of Urology, La Paz University Hospital, Madrid, Spain; p Department of Urology, Institute Mutualiste Montsouris, Paris, France; q Department of Urology, Hospital Cardiopulmonar, Salvador, Brazil; r Department of Urology, University of Patras, Patras, Greece; s Department of Urology, University Hospital Southampton NHS Trust, Southampton, UK; t Department of Urology, Hospital Clinico San Carlos, Madrid, Spain; u Department of Urology, Amsterdam UMC, Cancer Center Amsterdam, University of Amsterdam, Amsterdam, The Netherlands

**Keywords:** Curriculum, Laparoscopy, Surgery, Training, Urology, Delphi consensus

## Abstract

**Background and objective:**

While programmes such as the European Basic Laparoscopic Urological Skills have made strides in foundational training, a significant gap exists for intermediate and advanced laparoscopy education. Our objective is to develop and validate the European laparoscopic intermediate urological skills (LUSs2) curriculum, which will establish uniformity in the training of urological laparoscopic procedures and facilitate proficiency among practitioners.

**Methods:**

The study combines a literature review, cognitive task analysis development by a steering group, and a two-round Delphi survey involving international experts in urological laparoscopy. Consensus was defined as agreement of ≥70% among experts. The survey included statements on various laparoscopic procedures, assessed on a Likert scale from 1 (strongly disagree) to 9 (strongly agree).

**Key findings and limitations:**

The Delphi process achieved consensus on 85% (235/275) of statements, indicating a strong agreement on the curriculum’s content. Areas covered include renal hilum dissection, major vessel injury management, enucleation and renorrhaphy, vesicourethral anastomosis, and pyeloplasty. Limitations include the nonsystematic nature of the literature review and potential biases inherent in expert-based consensus methods.

**Conclusions and clinical implications:**

The LUSs2 curriculum significantly advances the standardised training of laparoscopic urological skills. It offers a detailed, consensus-validated framework that addresses the need for uniformity in surgical education and aims to enhance surgical proficiency and patient care.

**Patient summary:**

This study presents the development of a new standardised training curriculum for urological laparoscopic surgery. We intend this curriculum to improve the quality of surgical training and ensure high-quality patient care.

## Take Home Message

The European laparoscopic intermediate urological skills curriculum LUSs2 is the first development of an intermediate laparoscopic curriculum in urology. The combined approach, uniting a cognitive task analysis and Delphi consensus, facilitated the creation of a robust and finely tuned surgical training curriculum with the collaborative spirit that dives into medical education led by experts.

## 1 Introduction

### 1.1 Laparoscopic urological training and curricula

Proficiency in minimally invasive surgery, particularly in laparoscopy, is crucial for attaining surgical excellence and ensuring the best patient outcomes in urology [1]. The field’s constant evolution, marked by new technologies and surgical techniques, necessitates adaptive training methodologies. The European School of Urology’s (ESU’s) introduction of the European Basic Laparoscopic Urological Skills programme in 2011 was a significant step in this direction, but the lack of established and internationally validated curricula for intermediate and advanced laparoscopic skills is evident [2].

### 1.2 Benefits of standardised surgical training

The traditional Halsted model of “see one, do one, teach one” is increasingly being replaced by preoperative training models, including online materials, workshops, and training models [1]. Simulation-based training, common in high-risk professions, is particularly beneficial for rehearsing complex surgeries, allowing for skill refinement before the actual operations. This approach aligns with technological advancements and addresses the urgent need for standardised training methodologies in laparoscopic urology.

### 1.3 Lack of curricula and standardised training

Despite laparoscopy’s recognised role in urology, disparities in training, particularly in laparoscopic exposure, are evident [3], [4]. These disparities are more pronounced in certain regions, highlighting geographical inconsistencies in training standards [3], [4], [5]. The absence of advanced curricula underscores the need for a more structured approach to training in complex laparoscopic procedures.

### 1.4 Importance of consensus for surgical training

Establishing a consensus on training protocols, particularly with simulation models, is crucial [1]. A cognitive task analysis (CTA), developed initially in the military, offers a methodology for deconstructing the cognitive processes in each critical phase of a procedure [6]. Evidence suggests that a CTA-based instructional approach is superior in cultivating procedural knowledge and technical skills to conventional methods [7], [8], [9]. This study aims to develop and validate an intermediate laparoscopic urological skills curriculum to establish uniformity and facilitate proficiency in commonly performed urological laparoscopic procedures.

## 2 Methods

### 2.1 Literature review

A comprehensive nonsystematic literature review was performed according to the Preferred Reporting Items for Systematic Reviews and Meta-analyses (PRISMA) guidelines searching in PubMed/Medline, Embase, and Cochrane Library (CENTRAL and CDSR), using the combination of following keywords: “laparoscopy urology training”, “laparoscopy urology standardization”, “laparoscopy urology complications”, “renal hilum dissection”, “major vessel injury”, “renal tumour enucleation”, “laparoscopy vesicourethral anastomosis technique”, and “pyeloplasty”. The findings of the reviews provided the basis for the statements developed for voting in the Delphi survey and consensus meeting.

### 2.2 CTA development

A steering group of urologists involved in educational and surgical simulation activities (P.Z., T.R.O., G.P., J.G.R., H.d.V., C.B., W.B., T.S., M.R.S., and D.M.C.) within the European Association of Urology (EAU) and the ESU embarked on a series of collaborative meetings to meticulously craft a laparoscopic intermediate urological skills (LUSs2) curriculum using an in-depth CTA methodology. This curriculum involved intricate dissection of five distinct CTAs, meticulously outlining the sequential stages and essential equipment for specific tasks integral to the key laparoscopic urological surgical procedures. The specific tasks encompassed renal hilum dissection, major vessel injuries (MVIs), enucleation and renorrhaphy, vesicourethral (VU) anastomosis, and pyeloplasty.

These CTAs were initially drafted by renowned experts (renal hilum dissection: A.S., B.P., and D.V.; MVI: D.V., A.S., and F.G.; enucleation and renorrhaphy: D.V., A.S., and G.P.; VU anastomosis: R.R.T.-B., R.S.-S., and M.A.-M.; and pyeloplasty: A.S., M.A.-M., and P.Z.), with the final objective to be replicated within a hands-on training framework in simulators for training and evaluation. All steering group members meticulously reviewed and adapted all CTAs before their formal approval.

### 2.3 Two-round Delphi survey

Upon the competition and endorsement of the LUSs2 curriculum, our focus shifted to validation, prompting us to engage an array of international experts using the Delphi methodology. All CTAs were transformed into Delphi statements, facilitated by the online software Welphi (Welphi.com; Decision Eyes, Lisbon, Portugal). This process involved the creation of five different questionnaires, each aligned with a specific CTA, accompanied by a space for comments in each statement.

We employed a Likert scale spanning the spectrum from 1 to 9. A score of 1 represented “strongly disagree”, while 9 indicated “strongly agree”. We classified scores of 7, 8, and 9 as indicating agreement; 1, 2, and 3 as indicating nonagreement; and 4, 5, and 6 as denoting uncertainties.

The steering group agreed to define consensus as statements achieving ≥70% agreement (scores 7–9) and <10% disagreement (scores 1–3). Conversely, nonconsensus was defined when statements had ≥70% disagreement (scores 1–3) and <10% agreement (scores 7–9). The decision to use a 70% threshold was based on previous studies and research on consensus methods [10], [11], [12].

Experienced urologists in laparoscopic surgery from various sections of the EAU and different countries were invited via e-mail to participate in the Delphi consensus. To ensure the integrity of the process, participants’ identities and responses were protected, ensuring anonymity throughout the entire procedure. An experienced urologist in laparoscopic surgery was defined as a urologist with experience in the field of urological laparoscopy with significant hands-on experience with >5 yr of experience and >100 laparoscopic procedures per year, or >10 yr of experience with >50 laparoscopic procedures per year.

The first round was conducted from April 25 to May 25, 2023. The steering group analysed the results and comments, revising and resending the statements that did not reach an agreement. A second round was planned for these statements, involving a thorough review of comments and refinement to facilitate potential consensus.

The second round took place from June 26 to July 31, 2023. During this round, experts were informed of the consensus reached in the first round and the revised statements, including those with comments or edits as decided by the steering group.

### 2.4 Consensus meeting

Following the two rounds of the Delphi survey, statements that did not achieve consensus were presented for a vote to the expert panel during an online consensus meeting on November 22, 2023. The invited experts participated, where statements were subjected to a simple vote to determine “agreement” or “disagreement” based on the previously established >70% threshold.

## 3 Results

### 3.1 Literature review

The following question was reviewed: What is the current status of laparoscopy in urology in terms of training, standardisation, complications, and specific techniques such as renal hilum dissection, main vessel injuries, renal tumour enucleation, laparoscopic VU anastomosis, and pyeloplasty?

**Fig. 1:**
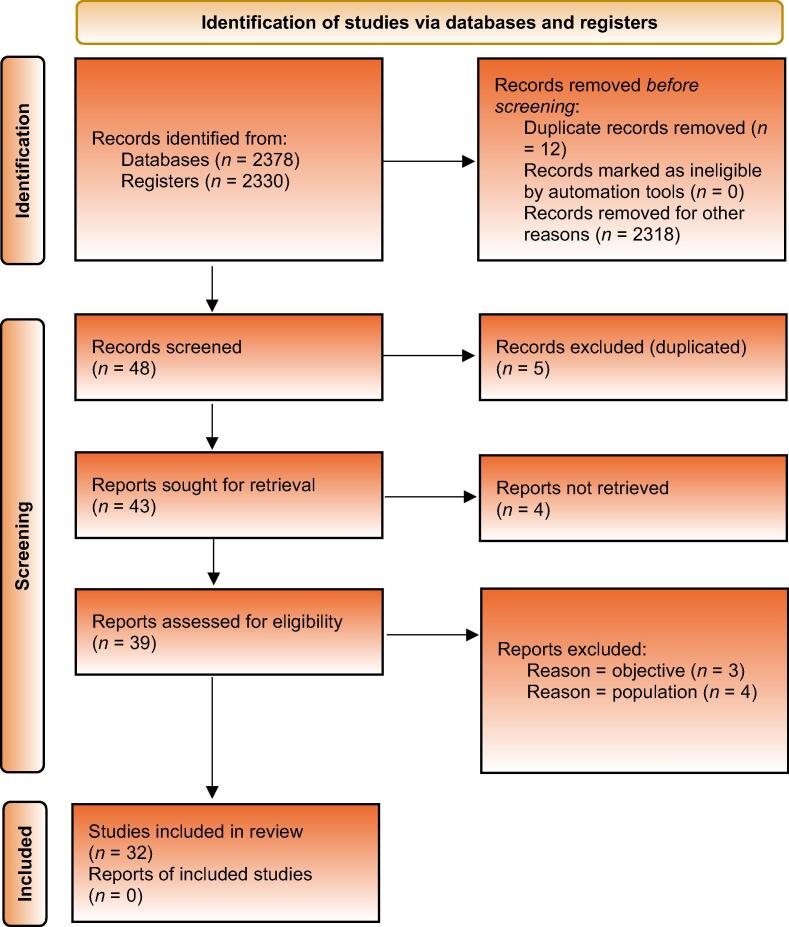
Preferred Reporting Items for Systematic Reviews and Meta-analyses (PRISMA) flowchart.

The selection process is outlined in the PRISMA diagram shown in Figure 1. The initial search reached 2330 records. Based on the title and abstract, 48 studies were screened, 39 were analysed in full text, and at the end of the process, 32 studies with reports were included in the qualitative analysis [13], [14], [15], [16], [17], [18], [19], [20], [21], [22], [23], [24], [25], [26], [27], [28], [29], [30], [31], [32], [33], [34], [35], [36], [37], [38], [39], [40], [41], [42], [43], [44]. Among these, 24 randomised controlled trials were identified [14], [15], [16], [17], [19], [22], [23], [24], [26], [27], [28], [29], [30], [31], [32], [33], [34], [37], [38], [39], [41], [42], [43], [44], and the included articles were used as a basis for the drafting of the statements. Twelve studies on laparoscopy urology training [13], [14], [15], [16], [17], [18], [19], [20], [21], [22], [23], [24], nine on laparoscopy urology standardisation [25], [26], [27], [28], [29], [30], [31], [32], [33], three on laparoscopy urology complications [34], [35], [36], three on renal tumour enucleation [37], [38], [44], and five on pyeloplasty [39], [40], [41], [42], [43] were identified. Articles used to define the consensus rules were not considered for analysis.

The literature also discusses various training methods for residents and novices, such as box trainers and simulators. Studies suggest that practice through structured training can lead to skill retention and potentially improve proficiency. However, the optimal method and the extent to which such training translates into clinical practice remain subjects of on-going research and debate.

It is evident that while the steep learning curve and effort required to achieve proficiency in laparoscopic surgery are recognised, the approaches to training vary significantly, and there is a persistent call for standardisation and improvement in training programmes.

### 3.2 Development of CTAs and Delphi statements

The steering group identified five complex laparoscopic tasks described in five different CTAs (Supplementary material). The essential key cognitive steps and decision-making processes of all CTAs were formulated into clear and concise Delphi statements so that the experts could evaluate and provide feedback on these. The statements were organised in a structured manner that reflected the sequence of cognitive processes involved in each task.

### 3.3 Two-round Delphi survey results

**Fig. 2:**
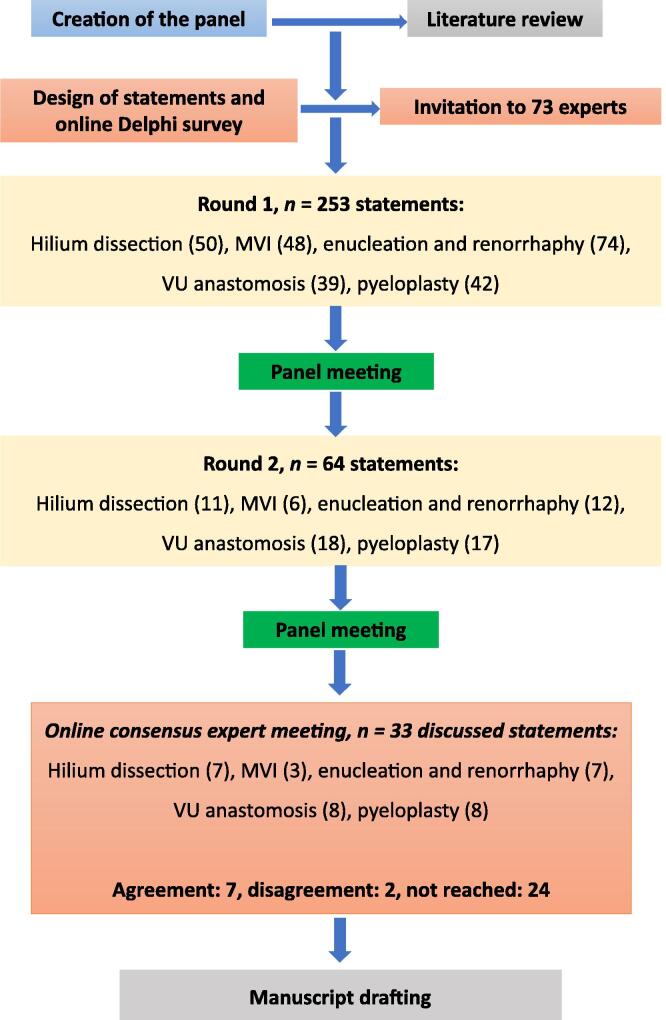
Delphi consensus study flowchart. MVI = major vessel injury; VU = vesicourethral.

Figure 2 shows the Delphi consensus flowchart for the survey. Table 1 presents the characteristics of the respondents. On average, 61 experts from 17 countries participated in the Delphi consensus, including Belgium, the Netherlands, Italy, Portugal, Germany, Spain, Greece, Turkey, and the UK. The overall response rate was 84%. A detailed breakdown of the participant numbers in each Delphi round can be found in Supplementary Table 1.

**Table 1:** Characteristics of the participants

Characteristics of the participants	
Age (yr), mean ±	47.15 ± 8.18
Region/country, *n* (%)	
Italy	16 (25.81)
Spain	12 (19.35)
The Netherlands	10 (16.13)
Portugal	4 (6.45)
Romania	3 (4.84)
Greece	3 (4.84)
UK	3 (4.84)
Germany	2 (3.23)
France	2 (3.23)
Belgium	1 (1.61)
Canada	1 (1.61)
Georgia	1 (1.61)
Switzerland	1 (1.61)
Turkey	1 (1.61)
UAE	1 (1.61)
USA	1 (1.61)
Years of practice, *n* (%)	
1–5	6 (9.68)
6–10	11 (17.74)
11–15	16 (25.81)
16–20	14 (22.58)
>20	15 (24.19)

In total, 205, 28, and two statements reached consensus in round 1, round 2, and the consensus meeting, respectively. Overall, 235 out of 275 (85%) statements reached an agreement, and 0.7% disagreed.

#### 3.3.1 Part I: hilum dissection

In this section, 38 of 50 (78%) statements reached agreement in the first round and four more in the second round, six statements were edited, and in the end, seven statements were discussed at the consensus meeting. The summary of statements regarding renal hilum dissection is shown in Table 2. There was an agreement regarding the usefulness of equipment such as atraumatic graspers, Maryland dissecting forceps, bulldog clamps, Hem-O-Lok clips, and suction-irrigation devices. There was also agreement that dissection can be considered complete when each vessel is sufficiently freed to safely place three Hem-O-Lok clips, a bulldog clamp, or tourniquets (in case of partial nephrectomy under warm ischaemia) with or without preservation of the adrenal gland, depending on the procedure. In the retroperitoneal approach, the Gaur balloon could be useful to create the operative field; the renal artery is the first to be identified, the psoas is the constant anatomical landmark, and the identification of the vena cava/aorta will help in easier finding of the hilum. There was no consensus on the imperative need to have instruments such as Satinsky clamp, Crawford clamp, EndoGia stapler, clips, or blood vessel sealing devices available.

**Table 2:** Summary of the statements regarding hilum dissection that were discussed and voted in round 1, round 2, and consensus meeting

Round 1					Round 2					Consensus meeting		
Statements round 1	Disagree %	Uncertain %	Agree %	Status	Statements round 2	Disagree %	Uncertain %	Agree %	Status	Disagree %	Agree %	Status
	1–3	4–6	7–9			1–3	4–6	7–9				
*Hilum dissection equipment*					*Hilum dissection equipment*							
1. A 5-mm atraumatic grasping forceps	8	16	76	Agreement								
2. A 5-mm straight (Maryland) dissecting forceps	13	25	64	Not reached	2. pair of A 5-mm straight (Maryland) dissecting forceps	5	17	80	Agreement			
3. A 5- or 10-mm right-angle dissecting forceps	10	15	77	Agreement								
4. A 5-mm bipolar dissecting forceps	5	17	78	Agreement								
5. A 5-mm blood vessel sealing device	18	22	61	Not reached	5. A blood vessel sealing device	16	16	69	Not reached	41	59	Not reached
6. Monopolar or bipolar laparoscopic scissors	16	16	69	Not reached	6. Laparoscopic scissors	9	11	80	Agreement			
7. A 5-mm endoclip applier and 5-mm clips	32	26	42	Not reached	7. A 5-mm endoclip applier and 5-mm clips are needed if radical nephrectomy has to be performed	40	25	35	Not reached	65	29	Not reached
8. A 10-mm endoclip applier and 10-mm clips	20	20	60	Not reached	8. A 10-mm endoclip applier and 10-mm clips are needed if radical nephrectomy has to be performed	14	10	76	Not reached	29	59	Not reached
9. Vessel loops (two different colours) for artery and vein occlusion if partial nephrectomy	17	23	60	Not reached	9. Vessel loops for artery and/or vein occlusion are recommended if partial nephrectomy has to be performed	10	17	73	Agreement			
10. Two long (10 cm) and two short (6 cm) pieces of a silicone 10F catheter to use as tourniquets if partial nephrectomy	31	37	32	Not reached	10. Two long (10 cm) and two short (6 cm) pieces of a silicone 10F catheter to use as tourniquets if partial nephrectomy has to be performed	31	43	25	Not reached	29	59	Not reached
11. Suction—irrigation device	8	5	87	Agreement								
12. A bulldog clamp applier if partial nephrectomy with clamping	6	3	92	Agreement								
13. Bulldog 5 mm straight and curved clamps if partial nephrectomy and clamping are required	7	27	67	Not reached	13. Bulldog 5-mm straight and curved clamps if partial nephrectomy and clamping are required	4	10	87	Agreement			
14. Bulldog 10 mm straight and curved clamps if partial nephrectomy with clamping	6	13	82	Agreement								
15. Satinsky laparoscopic clamp	28	34	38	Not reached	15. Satinsky laparoscopic clamp	25	42	33	Not reached	41	47	Not reached
16. Crawford laparoscopic curved clamp	29	41	30	Not reached	16. To have a Crawford laparoscopic curved clamp	27	52	21	Not reached	59	29	Not reached
17. Hem-O-Lok clips XL (in case of radical nephrectomy)	6	7	88	Agreement								
18. EndoGia stapler with a 45–60 mm vascular cassette in case of radical nephrectomy	34	22	43	Not reached	18. EndoGia stapler with a 45–60 mm vascular cassette in case of radical nephrectomy	42	22	37	Not reached	53	35	Not reached
*Hilum dissection approach*					*Hilum dissection approach*							
1. Always check images on CT or MRI first. Identify anatomy, best possible approach to the hilum, number of arteries/veins, and possible abnormalities/variations	0	0	100	Agreement								
2. Laparoscopic hilum dissection can be done with transperitoneal approach	0	0	100	Agreement								
3. Laparoscopic hilum dissection can be done by retroperitoneal approach	0	5	96	Agreement								
4. In the transperitoneal approach, the optimal way to perform dissection of structures is by an ascending route	6	15	82	Agreement								
5. Descendant or direct dissection is an alternative to the ascendant approach	7	10	85	Agreement								
*Hilum dissection transperitoneal approach round 1*					*Hilum dissection transperitoneal approach round 2*							
1. Rise up the lower kidney pole with the nondominant hand along with the ureter and gonadal vein (left side), to expose the psoas muscle and allow easier dissection of the fatty tissue	0	10	90	Agreement								
2. Apply vertical dissection to the fatty tissue overlying the psoas muscle	7	14	80	Agreement								
3. While dissecting the fatty tissue, the nondominant hand is progressively repositioned, moving cranially	0	7	94	Agreement								
4. The ureter and gonadal vein should be lifted up to allow easier and faster identification of the pedicle	6	11	85	Agreement								
5. Cut the connective tissue, even without cauterisation	11	25	64	Not reached								
6. Use cauterisation when cutting adhesions, after a wide dissection	2	13	86	Agreement								
7. To ensure controlling the hilum before it bifurcates proximally to the kidney, it can be useful to move medially and search for the cava/aorta	2	10	89	Agreement								
8. During vertical dissection of the perihilar fat, the renal vein is the first to show up in the majority of cases	0	2	99	Agreement								
9. When the renal vein is first seen, its surface has to be considered as the new cleavage plane to be followed in order to take apart the fatty tissue and achieve a full vascular exposure	0	6	93	Agreement								
10. A Maryland dissector can help in case of adhesions or a tight/absent cleavage plane over the vessels	5	25	70	Agreement								
11. Once the medial face of the renal vein is exposed fully, the posterior face can be freed with an angled dissector or (in case it is not available) the Maryland dissector. Any curved instrument might be useful	2	14	85	Agreement								
12. The renal artery is usually located posteriorly and cranially to the renal vein	4	8	89	Agreement								
13. Check for aberrant arteries or arterial branches when you find the main renal artery, or when you work closely to the kidney and not on the level of the cava or aorta	0	6	96	Agreement								
14. In order to facilitate the exposure of the renal artery, remember to perform effective lifting of the kidney with the nondominant hand	0	7	93	Agreement								
15. Special care has to be taken during the dissection of the renal artery as the surrounding fat may contain small vessels that could be damaged by excessive tension	2	2	97	Agreement								
16. In order to complete the dissection of the renal artery (posterior face), place an angled dissector between this and the vein, and open it slowly on its back	4	14	83	Agreement								
17. The renal artery can eventually be marked by placing a vessel loop (when needed or when performing a partial nephrectomy)	7	10	85	Agreement								
18. The dissection can be considered completed when each vessel is freed enough to safely place 3 clips, a bulldog clamp, or tourniquets (in case of a partial nephrectomy under warm ischaemia)	0	4	97	Agreement								
19. Depending on the procedure (adrenal sparing or not), the vein might be clipped and cut above the adrenal branch (adrenal sparing) or below the adrenal branch when performing a radical nephrectomy	4	5	92	Agreement								
*Hilum dissection retroperitoneal approach variations*					*Hilum dissection retroperitoneal approach variations*							
1. Expand the Gaur balloon in the retroperitoneal space to create the operating field	0	11	89	Agreement								
2. The lower kidney pole will be identified covered with the peri- and pararenal fat	0	14	86	Agreement								
3. Lift up the lower kidney pole along with the ureter to gain access to the hilum	2	13	86	Agreement								
4. Identification of the vena cava/aorta will help in an easier find of the hilum	2	17	81	Agreement								
5. In the retroperitoneal approach, the renal artery is identified first, as the pedicle is dissected from the posterior side	0	7	94	Agreement								
6. The renal artery is posterior, and the renal vein is anterior and usually caudal (inferior) to the renal artery	4	5	92	Agreement								
7. Before beginning dissection on the renal artery or vein, the horizontal positions of the major vessels (aorta on the left side, vena cava on the right: both parallel to the psoas) and vertical pulsations of the fat-covered renal artery laterally are looked for, and almost always visualised	2	11	87	Agreement								
8. One must remember that during renal retroperitoneoscopy, the psoas is the constant anatomic landmark.	0	5	95	Agreement								

CT = computed tomography; MRI = magnetic resonance imaging.

#### 3.3.2 Part II: MVI

Of the 48 statements, 44 (91%) reached agreement in the first round, 1 was edited for round 2, and two more statements were added; three statements reached agreement in round 2, and finally, three that did not reach agreement were discussed at the consensus meeting (Table 3).

**Table 3:** Summary of the statements about the assessment, handling, and repair of venous or arterial lesions (MVI) voted in round 1, round 2, and consensus meeting

Round 1					Round 2					Consensus meeting		
Statements round 1	Disagree %	Uncertain %	Agree %	Status	Statements round 2	Disagree %	Uncertain %	Agree %	Status	Disagree %	Agree %	Status
	1–3	4–6	7–9			1–3	4–6	7–9				
*MVI equipment*					*MVI equipment*							
1. Having a laparoscopic Satinsky clamp prepared is recommended	6	17	78	Agreement								
2. Having a laparoscopic Crawford clamp prepared is recommended	13	28	59	Not reached	2. Having a laparoscopic Crawford clamp prepared is recommended	11	15	75	Not reached	11	89	Agreement
3. Having laparoscopic clips with an applier prepared is recommended	5	10	86	Agreement								
4. Having Hem-O-Lok clips (sizes M-L, L, and XL) prepared is recommended	7	9	86	Agreement								
5. 4-0 or 5-0 Prolene sutures should be ready	0	3	98	Agreement								
6. Pre-prepared 4-0 or 5-0 Prolene sutures with a knot at the tail and a Hem-O-Lok or Lapra-Ty clip should be ready (rescue stitch)	6	8	87	Agreement								
7. Two needle holders should be prepared	7	15	78	Agreement								
8. A closed suction drain should be left at the end of a surgery that required laparoscopic MVI repair	10	26	65	Not reached	8.1. A closed suction drain should be left at the end of a surgery that required MVI repair	22	18	61	Not reached	39	61	Not reached
					8.2. A drain should be left at the end of a surgery that required MVI repair	8	8	85	Agreement			
					8.2. A passive drain should be left at the end of a surgery that required MVI repair	8	15	78	Agreement			
9. Bipolar energy devices should be prepared in case of MVI repair	9	17	75	Agreement								
10. Ultrasound energy devices should be prepared in case of MVI repair	19	38	43	Not reached	10. Ultrasound energy devices should be prepared in case of MVI repair	17	34	49	Not reached	61	39	Not reached
*MVI repair approach*					*MVI repair approach*							
1. While experiencing a major vein injury, a quick decision should be made between ligating the vessel immediately or continuing dissection to allow better exposure	5	11	84	Agreement								
2. While experiencing a major artery injury, a quick estimation of the injury to the circumference should be done	4	7	90	Agreement								
*MVI repair: handling and repair of venous injuries*					*MVI repair: handling and repair of venous injuries*							
1. One of the first steps to control a major vein injury is compression with a gauze or clamp with an atraumatic grasper	0	3	97	Agreement								
2. One of the first steps to control a major vein injury is to increase the pneumoperitoneum pressure to gain more time (be aware that in case of large injuries to vena cava, it is not advisable to increase pressure, as this could lead to gas embolism)	5	12	84	Agreement								
3. Avoid further damage and remove scissors	8	14	78	Agreement								
4. Avoid further damage and avoid applying clips without proper exposure to the bleeding vessel	0	2	98	Agreement								
5. Immediately communicate with your assistant and localise the injured vein together	0	3	98	Agreement								
6. The assistant should be aware of having the optic lens clean and away from the bleeding points	0	0	100	Agreement								
7. The assistant should be aware of applying the correct amount of pressure with the suction device	0	2	99	Agreement								
8. Communicate with the anaesthesiologist about the injury to estimate the amount of blood loss	0	2	99	Agreement								
9. Communicate with the scrub nurse to prepare an open surgery tray	0	4	97	Agreement								
10. Communicate with the scrub nurse to prepare extra trocars, needle drivers, Prolene sutures	0	0	100	Agreement								
11. Plan your next steps beforehand and make sure that the entire team understands your plan and will follow it	0	0	101	Agreement								
12. Increase gas flow to a high level (40 l/min) and pressure up to 20 mmHg (especially if you need aggressive suctioning) to gain exposure and reduce venous leakage (be aware that in case of large injuries to vena cava, it is not advisable to increase pressure, as this could lead to gas embolism)	2	14	84	Agreement								
13. Identify the injury and explore surrounding structures to allow complete visualisation of the region	0	0	99	Agreement								
14. For small tears in the vena cava, local pressure plus haemostatic agent application should be enough	2	18	81	Agreement								
15. In case of a small venotomy, after adequate exposure, perform a figure-8 stitch with a Hem-O-Lok or Lapra-Ty on one side of the knot (rescue stitch)	5	14	81	Agreement								
16. In case of a small venotomy, after adequate exposure, perform a figure-8 stitch using a suture with freehand knot tying (rescue stitch)	0	17	83	Agreement								
17. In case of a large injury, consider a temporal clamp of the vessel proximal and distal to the injury	0	2	98	Agreement								
18. Useful instruments for vessel clamping in large venous injuries are Satinsky clamp (12 mm trocar), curved Crawford clamp (10 mm trocar), and bulldog clams (10–12 mm trocars)	0	6	95	Agreement								
19. After clamping a vessel in large venous injuries, consider conversion to open surgery if you find a large defect hard to manage laparoscopically	0	4	97	Agreement								
20.In case of large venous injuries, if you find it possible, repair the injury with interrupted 4-0 or 5-0 Prolene suture	7	4	91	Agreement								
21. It is important to ask the anaesthesiologist to temporarily lower blood pressure to 60/70 mmHg (minimal pressure to guarantee regular renal and cerebral perfusion) to allow easier control of the injury, if possible	2	14	85	Agreement								
*MVI repair: handling and repair of arterial injuries*					*MVI repair: handling and repair of arterial injuries*							
1. Arterial injuries need suturing, grafting, end-to-end anastomosis, or bypassing depending on the extent of the injury	2	9	90	Agreement								
2. Arterial lacerations need suturing with 4-0 or 5-0 Prolene suture (simple or figure-8 stitch)	0	9	91	Agreement								
3. For injuries that encompass >30% of the circumference of the artery, a repair with vein grafts or Gore-Tex patch graphs is needed	2	31	67	Not reached	3. For injuries that encompass >30% of the circumference of the artery, a repair with vein grafts or Gore-Tex patch graphs is needed	2	19	79	Agreement			
4. For injuries that encompass >30% of the circumference of the artery, ask for immediate vascular surgery assistance	4	5	92	Agreement								
5. Complete arterial transection requires end-to-end anastomosis	0	16	85	Agreement								
6. More complicated arterial injuries require bypassing vascular surgery	0	16	85	Agreement								
7. An arterial repair with vein grafts or Gore-Tex patch requires mobilisation of the artery and clamping above and below the level of the injury (with either laparoscopic bulldog clamps or vessel loop tourniquets)	0	8	92	Agreement								
8. To perform an end-to-end anastomosis in complete arterial transection, mobilisation of the artery and clamping above and below the level of the injury (with either laparoscopic bulldog clamps or vessel loop tourniquets) is required	0	4	97	Agreement								
9. Complex injuries that require bypassing vascular surgery require mobilisation of the artery and clamping above and below the level of the injury (with either laparoscopic bulldog clamps or vessel loop tourniquets)	0	10	90	Agreement								
10. A heparin flush should be done before closing arterial defects	0	19	82	Agreement								
11. In any of the steps mentioned, vascular surgeon consultation may be needed	2	6	95	Agreement								
*MVI repair: urgent conversion to open surgery*					*MVI repair: urgent conversion to open surgery*							
1. In case of (laparoscopic) uncontrollable bleeding, a prompt decision to convert to open surgery should be made	0	2	99	Agreement								
2. Iodine and sterile cover should already be anticipated on a possible conversion before starting a laparoscopic procedure	5	12	83	Agreement								
3. The patient should preferably be moved to a supine position, and a midline incision should be made for adequate exposure of major vessels depending on the surgeon's preference	6	15	79	Agreement								
4. In case of an injury with the patient in the lateral decubitus, patient should preferably be moved to the supine position to perform a subcostal (Chevron) incision for adequate exposure of major vessels depending on the surgeon's preference	6	23	71	Agreement								

MVI = major vessel injury.

In case laparoscopic MVI repair is needed, it is recommended that a laparoscopic Satinsky clamp, laparoscopic Crawford clamp, Hem-O-Lok clips (sizes M-L, L, and XL), two needle holders, and bipolar energy devices be prepared.

The statement regarding a closed suction drain to be left at the end of the surgery did not reach agreement in any of the rounds or consensus meetings. For round 2, two more statements were added and approved: one about leaving a drain and the other about leaving a passive suction drain.

We consider these useful advice for laparoscopists. However, there was no consensus about the need for prepared ultrasound energy devices or leaving a suction drain at the end of MVI repair.

#### 3.3.3 Part III: enucleation and renorrhaphy

Seventy-four statements were drafted initially for this section, 67 reached consensus in round 1 (90%), five were edited, and five were added for round 2. In round 2, five statements reached an agreement, and seven were discussed in the consensus meeting.

There was consensus on the usefulness and availability of equipment such as bipolar forceps, suction-irrigation devices, monopolar scissors, needle holders, and bulldog forceps or tourniquets. In addition, there is consensus that laparoscopic enucleation and renorrhaphy can be performed transperitoneally or retroperitoneally. The preferred route should be chosen according to the location of the renal lesion and the surgeon's experience. Zero ischaemia, warm ischaemia, selective/superselective clamping, and early arterial unclamping are viable options. Furthermore, monofilament suture is recommended for inner renorrhaphy, while no consensus was obtained for the type of suture for the outer renorrhaphy. Statements about the exposure technique, tumour excision, and renorrhaphy techniques are given in Table 4.

**Table 4:** Summary of the statements about the equipment, exposure technique, tumour excision, and renorrhaphy techniques for enucleation and renorrhaphy voted in round 1, round 2, and consensus meeting

Round 1					Round 2					Consensus meeting		
Statements round 1	Disagree %	Uncertain %	Agree %	Status	Statements round 2	Disagree %	Uncertain %	Agree %	Status	Disagree %	Agree %	Status
	1–3	4–6	7–9			1–3	4–6	7–9				
*Enucleation and renorrhaphy equipment*					*Enucleation and renorrhaphy equipment*							
1. A 2 × 10–12 mm trocar is needed	3	14	82	Agreement								
2. A 2–3 × 5 mm trocar is needed	9	15	77	Agreement								
3. A 1 × laparoscopic 5 mm bipolar grasper is needed	2	20	79	Agreement								
4. A 1 × laparoscopic 5 mm aspiration-irrigation device is needed	0	0	99	Agreement								
5. A pair of 1 × laparoscopic monopolar scissors is needed	2	7	93	Agreement								
6. A 1 × laparoscopic 5 or 10 mm right-angle dissecting forceps is needed	19	24	56	Not reached	6. A 1 × laparoscopic 5 or 10 mm right-angle dissecting forceps is needed	24	9	67	Not reached	42	58	Not reached
7. A 1 × laparoscopic 5 mm Maryland forceps is needed	9	24	66	Not reached	7.1. A 1 × laparoscopic 5 mm Maryland forceps is needed	7	22	71	Agreement			
					7.2. A 1 × laparoscopic fenestrated grasper is needed	12	21	68	Not reached	21	79	Agreement
8. A 1 × laparoscopic 10-mm Satinsky or bulldog clamp applier is needed	8	6	85	Agreement								
9. 2 × laparoscopic 5 mm needle holders are needed	10	8	81	Agreement								
10. A 1 × laparoscopic probe for intraoperative ultrasound is needed	5	32	62	Not reached	10. A 1 × laparoscopic probe for intraoperative ultrasound is needed for the evaluation of the tumour and objectifying deepness during laparoscopic enucleation and renorrhaphy	0	27	73	Agreement			
11. A 2 × bulldog clamp or tourniquet is needed	4	10	88	Agreement								
12. a 30° optical lens camera on the optic trocar is suggested (it could be related to the transperitoneal or retroperitoneal approach)	7	12	82	Agreement								
13. Optional: advanced sealing system can be helpful	8	21	70	Agreement								
14. Barbed sutures are needed	20	20	60	Not reached	14.1. Barbed sutures are recommended for inner renorrhaphy	16	16	69	Not reached	58	42	Not reached
					14.2. Barbed sutures are recommended for outer/capsular renorrhaphy	16	27	55	Not reached	47	53	Not reached
					14.3. Polyglactin (Vicryl) is recommended for inner renorrhaphy	16	23	59	Not reached	63	37	Not reached
					14.4. Polyglactin (Vicryl) is recommended for outer/capsular renorrhaphy	13	27	59	Not reached	47	53	Not reached
					14.5. Monofilament is recommended for inner renorrhaphy	7	16	78	Agreement			
15. CT-1, 36, 1/2C, Taperpoint, 0, polyglactin (or monofilament PDS) 20–25 cm is needed	10	22	68	Not reached	15. Monofilament is recommended for outer/capsular renorrhaphy	30	29	41	Not reached	26	74	Agreement
16. CT-2, 26, 1/2C, Taperpoint, polyglactin (or monofilament PDS), 2/0, 20 cm is needed	2	27	72	Agreement								
17. Nonabsorbable or absorbable clips and clip applier are needed for laparoscopic enucleation and renorrhaphy	0	5	95	Agreement								
18. Haemostatic agents in some cases are needed for laparoscopic enucleation and renorrhaphy	2	11	89	Agreement								
*Enucleation and renorrhaphy planning and approach*					*Enucleation and renorrhaphy planning and approach*							
1. Preoperative planning based on imaging is mandatory before laparoscopic enucleation and renorrhaphy to check dimension, shape, exophytic/endophytic proportions, distance from calyces, nearness to other structures, and other abnormalities	0	0	100	Agreement								
2. Laparoscopic enucleation and renorrhaphy can be done via a transperitoneal approach	0	0	99	Agreement								
3. Laparoscopic enucleation and renorrhaphy can be done via a retroperitoneal approach	0	9	91	Agreement								
4. The preferred approach should be chosen in accordance with the location of the renal lesion and the experience of the surgeon	0	0	100	Agreement								
5. Zero ischaemia is a viable option during laparoscopic enucleation and renorrhaphy	4	9	89	Agreement								
6. Warm ischaemia is a viable option during laparoscopic enucleation and renorrhaphy	0	2	99	Agreement								
7. Selective or superselective clamping (with or without fluorescence) is a viable option during laparoscopic enucleation and renorrhaphy	0	6	95	Agreement								
8. Early arterial unclamping is a viable option during laparoscopic enucleation and renorrhaphy	0	7	93	Agreement								
*Enucleation and renorrhaphy: tumour exposition and excision*					*Enucleation and renorrhaphy: tumour exposition and excision*							
1. Follow the same passage and technique of hilum dissection after thoroughly analysing the CT for arterial vasculature	0	5	96	Agreement								
2. Secure artery/arteries with a vessel loop with Hem-O-Lok	6	22	72	Agreement								
3. Open Gerota’s fascia near the location of the lesion	5	8	87	Agreement								
4. Make a first incision on the perirenal fat, near the location of the lesion	8	14	78	Agreement								
5. Follow the cleavage plane between capsule and fat until the border of the tumour/adipose tissue covering the tumour (if the tumour is exophytic)	0	3	97	Agreement								
6. In case of endophytic masses, the borders of the tumour are identified with US intraoperative guidance after defatting	0	4	97	Agreement								
7. Complete exposure and defatting of kidney surface except for fat overlying the tumour	0	6	93	Agreement								
8. Mark the resection line all around the tumour edge according to the endoscopic/intraoperative US appearance of the tumour	2	7	92	Agreement								
9. Mobilise the kidney as much as needed	2	4	95	Agreement								
10. Time out: take a minute to check that everything is ready: bulldog clamps or Rummel tourniquet, needle drivers, sutures (type and length, ready prepared), endoclips (Hem-O-Lok or similar)	0	2	99	Agreement								
11. In case of large masses, clamping can be applied after marking the resection line	0	0	100	Agreement								
12. In case of small masses, clamping can be evaluated during enucleation according to the surgeon's experience and ability to control bleeding adequately, thus providing a clean working area	3	2	95	Agreement								
13. Communicate with the anaesthesiologist for the ischaemia time; start and stop to be documented	0	0	99	Agreement								
14. Throughout the procedure, the assistant will provide a clean field by using suction, rinsing/flushing saline when needed, and/or pressing down any bleeding points	0	4	96	Agreement								
15. With nondominant hand gently lift the perirenal fat overlying the tumour, and with dominant hand make a sharp incision on the renal capsule 2/3 mm away from the border of the tumour	0	7	94	Agreement								
16. Widen the first sharp incision up to overall 5 mm, to allow easier identification of the tissues	0	11	88	Agreement								
17. Search for colour difference compared with the surrounding kidney parenchyma (whitish/yellowish), cleavable plane	0	9	92	Agreement								
18. Pay attention to complex cysts to avoid any type of traction, and generally an enucleoresection should be preferred in these cases	0	3	96	Agreement								
19. Produce countertraction between tumour (nondominant hand) and parenchyma (dominant hand) to identify and follow the correct cleavage plane, and to avoid inadvertent rupture of tumour pseudocapsule	0	2	98	Agreement								
20. In case of enucleation, the surgeon will provide dissection as close as possible to the tumour pseudocapsule by a blunt and sharp technique	2	0	99	Agreement								
21. Inadvertent cut or entry into the tumour should be recognised by the effluence of necrotic/oncotic material, and/or the more yellowish tissue emerging into the dissecting plane	0	7	94	Agreement								
22. Cutting the pelvicalyceal system will be recognised by the effluence of urine in the operating field	5	19	75	Approved								
23. In order to readjust the plane between the renal parenchyma and the tumour correctly, you should step back (with the camera and the instruments) a few millimetres from the wrong plane, identify the normal parenchyma, and dissect either superiorly or inferiorly to the correct plane	0	2	98	Agreement								
24. Visible bleeding vessels and incidental opening of the collecting system are ligated with running capsular suture with different stitches (polyglactin, CT-2, 26, 1/2C, taper-point needle, PDS, or Prolene)	4	17	80	Agreement								
25. Early arterial unclamping could be attempted at this time in order to decrease warm ischaemia time and help identify bleeding points	2	12	87	Agreement								
*Enucleation and renorrhaphy: renorrhaphy technique*					*Enucleation and renorrhaphy: renorrhaphy technique*							
1. To close the defect: one layer in cortical small defects	4	6	92	Agreement								
2. To close the defect: two layers in deeper defects (inner renorrhaphy for vessels)	2	15	84	Agreement								
3. To close the defect: three layers in which the collecting system is closed separately	9	25	67	Not reached	3. To close the defect: three layers in some particular cases with complex lesions and deep defect of parenchyma in which the collecting system is closed separately	4	16	80	Approved			
4. There are different renorrhaphy techniques	0	2	98	Agreement								
5. In some cases, the renorrhaphy could be avoided with a sutureless technique. In this situation, coagulation and biological haemostatic agents are used	13	20	68	Not reached	5. In some particular conditions (small masses, poorly vascularised), the renorrhaphy could be avoided with a sutureless technique. In this situation, coagulation and biological haemostatic agents are used	6	10	85	Approved			
6. A CT-1 ½ circle needle polyglactin/PDS suture (or barbed suture) is prepared on the back table by applying a Hem-O-Lok clip to the end with a prefixed knot at the free end of the suture, and the clip is applied just in front of the knot preventing slipping. The suture must be fixed exactly at the centre of the clip, and perpendicularly, this area exerts more firmly and distributes the force	0	12	88	Agreement								
7. (At the end of the external renorrhaphy) As the renal parenchyma is reapproximated, internal renorrhaphy may loosen. At this point, traction on both tails (one at a time) of the internal renorrhaphy line will expose the knot of the suture and the clip applied on each site	2	7	92	Agreement								
8. Another nonabsorbable clip is then applied above these (on its corner, we call it the “locking” nonabsorbable clip), in close proximity to the renal capsule, in order to reinforce the internal renorrhaphy	0	13	87	Agreement								
*Enucleation and renorrhaphy: running-sliding clip technique*					*Enucleation and renorrhaphy: running-sliding clip technique*							
1. The recommended length of the suture is 18–20 cm, depending on the size of the defect	0	12	89	Agreement								
2. Based on individual anatomy, two such sutures should be prefixed and ready for use	0	9	91	Agreement								
3. The suture is passed through the renal capsule at the edge of the nephrothomy, from the renal parenchyma outside to the renal “bed” inside. The renal bed is sutured over and over to seal any bleeding vessels or any opening of the pelvicalyceal system. The suture is finally pulled out from the renal bed inside to the renal parenchyma outside at the opposite edge of the nephrothomy	0	2	98	Agreement								
4. The suture line is locked by the appliance of a nonabsorbable clip at the exterior part of the suture in contact with the surface of the renal parenchyma	2	3	96	Agreement								
*Enucleation and renorrhaphy: interrupted mattress sliding technique*					*Enucleation and renorrhaphy: interrupted mattress sliding technique*							
1. The recommended length of the suture is 10–12 cm	3	22	74	Agreement								
2. Mattress sutures: The first suture is passed through the renal capsule at the edge of the nephrothomy, from the renal parenchyma outside to the renal “bed” inside. Next, a separate bite of the renal bed is taken, to seal any bleeding vessels or any opening of the pelvicalyceal system, and the suture is passed from inside out to the opposite site of the renal parenchyma. The thread of the suture is locked with a new Hem-O-Lok clip, but the suture is not tightened at this moment and left loose instead. Several such sutures are placed alongside the surface area needed to be reapproximated and remain loose	3	20	76	Agreement								
3. When all the sutures have been placed, start tightening them one by one by pulling the suture and pressing the Hem-O-Lok clip at the thread of the suture, and also at the tail of the suture against the renal parenchyma so as to reapproximate the inner parenchyma and “lock” the sutures. Repeat the same procedure for all the sutures of the internal renorrhaphy	5	17	78	Agreement								
*Enucleation and renorrhaphy: external renorrhaphy running-sliding technique*					*Enucleation and renorrhaphy: external renorrhaphy running-sliding technique*							
1. The recommended length of the suture is 18–20 cm	4	7	90	Agreement								
2. The suture is then passed through the renal capsule perpendicularly and pulled to the desired tension	2	7	92	Agreement								
3. A second Hem-O-Lok clip secures it snugly against the opposing renal capsule with the aid of a right-angle forceps	2	14	85	Agreement								
4. In preparation for the next throw, a new Hem-O-Lok clip is applied 1.5 cm proximal to the second set of clips	2	12	87	Agreement								
*Enucleation and renorrhaphy: external renorrhaphy interrupted mattress sliding technique*					*Enucleation and renorrhaphy: external renorrhaphy interrupted mattress sliding technique*							
1. The recommended length of the suture is 12–15 cm	2	16	83	Agreement								
2. The suture is then passed through the renal capsule perpendicularly and pulled to the desired tension	2	8	90	Agreement								
3. A second Hem-O-Lok clip secures it snugly against the opposing renal capsule with the aid of a right-angle forceps	2	16	82	Agreement								
4. The suture is then cut, the needle is removed, and another separate prefixed suture is passed through the renal capsule in a 3–5 mm width distance from the entrance of the previous suture	2	19	80	Agreement								

CT = computed tomography; USA = ultrasound.

#### 3.3.4 Part IV: VU anastomosis

Thirty-nine statements were initially proposed; 24 reached agreements (61%) in round 1. Four statements were edited for round 2, and three new statements were added. Round 2 included 18 statements, of which ten reached agreement. The remaining eight statements were discussed in the consensus meeting, and none reached an agreement.

Regarding the required equipment, it was agreed that at least one needle holder is recommended for laparoscopic VU anastomosis and one 18-20F Foley catheter is needed for laparoscopic VU anastomosis. In addition, two unidirectional or one bidirectional barbed suture may be useful for laparoscopic VU anastomosis. It was agreed that knots should be done and kept outside the urethral lumen, and to inflate the balloon and test the integrity of the anastomosis by filling the bladder with 150 ml saline is useful at the end of the procedure. The VU anastomosis technique statements are available in Table 5. There was no consensus on the ideal needle for VU anastomosis.

**Table 5:** Summary of the statements regarding vesicourethral anastomosis voted in round 1, round 2, and consensus meeting

Round 1					Round 2						Consensus meeting		
Statements round 1	Disagree %	Uncertain %	Agree %	Status	Statements round 2	Disagree %	Uncertain %	Agree %	Do not perform the procedure	Status	Disagree %	Agree %	Status
	1–3	4–6	7–9			1–3	4–6	7–9					
*Vesicourethral anastomosis equipment*					*Vesicourethral anastomosis equipment*								
1. One pair of monopolar scissors is needed	16	10	74	Not reached	1. One pair of monopolar scissors is recommended to cut the threads	8	5	87		Agreement			
2. Two needle holders are needed	13	14	72	Not reached	2. At least one needle holder is recommended	2	6	93		Agreement			
3. One 5 mm straight (Maryland) dissecting forceps is needed	9	21	70	Not reached	3. One 5 mm straight (Maryland) dissecting forceps is recommended	7	24	69		Not reached	56	44	Not reached
4. One 18-20 Fr Foley catheter is needed	0	5	96	Agreement									
					4.2. One fenestrated grasper is needed	24	33	44		Not reached	56	44	Not reached
5. Two twin 3-0 poliglecaprone-25 sutures on a CT-1 needle that are tied together on the two ends are needed	9	23	66	Not reached	5. Two twin 3-0 poliglecaprone-25 sutures that are tied together on the two ends are needed	7	14	78		Agreement			
6. Alternatively, two polyglactin sutures on a CT-1 needle that are tied together on the two ends are needed	11	26	62	Not reached	6. Two polyglactin sutures on a CT-1 needle that are tied together on the two ends are needed	10	15	74		Not reached	61	39	Not reached
7. Alternatively, two unidirectional or one bidirectional barbed suture is needed	4	3	94	Agreement									
					8. A suture with a 1/2 circle 26 mm needle is needed	10	30	60		Not reached	50	50	Not reached
					9. A suture with a 5/8 circle 26 mm needle is needed	15	25	59		Not reached	50	50	Not reached
*Vesicourethral anastomosis procedural step 1*					*Vesicourethral anastomosis procedural step 1*								
1. Prepare a 12–20 cm suture, depending on the width of the bladder neck	0	14	85	Agreement									
2. If the bladder neck is wide: start reconstruction by closing the two corners with a figure of 8 suture in order to create a fish mouth, or with an anterior or posterior bladder closure	5	12	82	Agreement									
3. Ask for a perineal push. The manoeuvre will expose urethral stump and urethral mucosa	0	15	84	Agreement									
4. Place both needles outside in through the bladder neck and inside out through the urethra, the right needle from the 5:30 o’clock towards the 3:00 o’clock position and the left needle from the 6:30 o’clock towards the 9:00 o’clock position	2	16	82	Agreement									
5. Pull the sutures with gentle traction on each thread (simultaneously or alternatively) in order to bring the bladder neck adjacent to the urethra without leaving a gap within the dorsal part of the anastomosis	0	8	92	Agreement									
6. Avoid tearing the urethra by pulling the suture gently, upwards or laterally, and having the suture pass between the two jaws of the open needle holder placed adjacent to the urethra	0	5	95	Agreement									
7. Place a 18-20 Fr Foley catheter into the bladder	5	8	86	Agreement									
8. Pass the sutures outside in on the bladder neck and inside out on the urethra, running from the 6:30 and 5:30 o’clock positions towards the 10:00 and 2:00 o'clock positions, respectively	0	11	89	Agreement									
9. Check the integrity of the ureteral orifice before start and avoid catching ureters in the bladder neck	0	4	97	Agreement									
10. After each urethral stitch, the catheter needs to be mobilised gently in order to rule out inadvertent fixation	2	7	92	Agreement									
11. Continue the sutures to the 12:00 o'clock position	0	3	97	Agreement									
12. Place a new 18-20 Fr Foley catheter through the anastomosis	2	19	79	Agreement									
13. Tie the sutures to each other so that the knot rests on the exterior of the bladder (unless a barbed suture is used, these do not require a knot to lock)	4	4	94	Agreement									
14. Perform an anterior tennis-racket suture if there is a mismatch between the bladder and the urethra	2	10	89	Agreement									
15. Inflate the balloon and test the integrity of the anastomosis by filling the bladder with 150 ml saline	0	9	90	Agreement									
*Vesicourethral anastomosis procedural step 2*					*Vesicourethral anastomosis procedural step 2*								
1. Prepare a 15 cm absorbable suture	5	25	70	Not reached	1. Prepare a 15-cm absorbable suture	1.8	20.2	77.7	8	Agreement			
2. Pass the needle at the 9:00 o’clock position outside in on the bladder neck and at the 9:00 o’clock position inside out on the urethra and tie the suture into a knot with the suture tail	7	23	72	Agreement									
3. Pass the needle at the 11:00 o’clock position outside in on the bladder neck and at the 7:00 o’clock position inside out on the urethra	9	24	68	Not reached	3. Pass the needle at the 11:00 o’clock position outside in on the bladder neck and at the 7:00 o’clock position inside out on the urethra	0	15.3	81.4	12	Agreement			
4. Pass the single suture continuously at the 1:00 o’clock position on the bladder neck, at the 5:00 o’clock position on the urethra, at the 1:00 o’clock position on the urethra, at the 7:00 o’clock position on the bladder neck, and at the 11:00 o‘clock position on the urethra	10	22	68	Not reached	4. Pass the single suture continuously at the 1:00 o’clock position on the bladder neck, at the 5:00 o’clock position on the urethra, at the 1:00 o’clock position on the urethra, at the 7:00 o’clock position on the bladder neck, and at the 11:00 o’clock position on the urethra	1.9	19.1	78.7	12	Agreement			
5. Place a 18-20 Fr Foley catheter into the bladder and tie the knot with the suture tail at the 9 o’clock position (unless a barbed suture is used, these do not require a knot to lock)	8	19	72	Agreement									
6. Inflate the balloon and test the integrity of the anastomosis by filling the bladder with 150 ml saline	4	14	82	Agreement									
*Vesicourethral anastomosis procedural step 3*					*Vesicourethral anastomosis procedural step 3*								
1. Prepare a 15 cm absorbable suture	6	27	66	Not reached	1. Prepare a 15 cm absorbable suture	1.8	18.4	79.6	8	Agreement			
2. Place the first suture inside out on the urethra and outside in on the bladder neck at the 5:00 o’clock position	13	29	58	Not reached	2. Place the first suture inside out on the urethra and outside in on the bladder neck at the 5:00 o’clock position	5.5	20.3	73.9	8	Agreement			
3. Tie the suture inside the urethral lumen	19	29	52	Not reached	3. Tie the suture inside the urethral lumen	16.3	25.4	58	7	Not reached	72	22	Not reached
4. Place the second suture inside out on the urethra and outside in on the bladder neck at the 7:00 o’clock position	12	36	54	Not reached	4. Place the second suture inside out on the urethra and outside in on the bladder neck at the 7:00 o’clock position	5.4	28.9	65.3	7	Not reached	33	61	Not reached
5. Tie the suture inside the urethral lumen	21	27	52	Not reached	5. Tie the suture inside the urethral lumen	19.9	18.1	61.7	7	Not reached	67	28	Not reached
6. Place four sutures symmetrically at the 4:00, 8:00, 2:00, and 10:00 o’clock positions outside in on the urethra and inside out on the bladder neck	6	27	66	Not reached	6. Place four sutures symmetrically at the 4:00, 8:00, 2:00, and 10:00 o’clock positions outside in on the urethra and inside out on the bladder neck	7.2	19.9	72.5	7	Agreement			
7. Tie the sutures outside the urethral lumen	8	21	71	Agreement									
8. Place the final 2 sutures outside in on the urethra and inside out on the bladder neck at the 11:00 and 1:00 o’clock positions	9	24	66	Not reached	8. Place the final 2 sutures outside in on the urethra and inside out on the bladder neck at the 11:00 and 1:00 o’clock positions	7.2	18	74.4	7	Agreement			
9. Place a 18-20 Fr Foley catheter into the bladder	6	13	81	Agreement									
10. Tie the final sutures outside the urethral lumen	6	16	78	Agreement									
11. Inflate the balloon and test the integrity of the anastomosis by filling the bladder with 150 ml saline	6	18	76	Agreement									

#### 3.3.5 Part V: pyeloplasty

Forty-two statements were drafted initially, 32 were approved in round 1 (76%), seven were edited, and ten were added for round 2; after round 2, six statements were approved, and ten were discussed in the consensus meeting (Table 6).

**Table 6:** Summary of the statements regarding pyeloplasty voted in round 1, round 2, and consensus meeting

Round 1					Round 2					Consensus meeting		
Statements round 1	Disagree %	Uncertain %	Agree %	Status	Statements round 2	Disagree %	Uncertain %	Agree %	Status	Disagree %	Agree %	Status
	1–3	4–6	7–9			1–3	4–6	7–9				
*Pyeloplasty equipment*					*Pyeloplasty equipment*							
1. A Gaur balloon dilator is needed if retroperitoneal access is planned	5	21	73	Agreement								
2. Veress or Hasson techniques should be used for transperitoneal access as well as direct vision trocar	9	9	84	Agreement								
3. Two 10 mm trocars are required	26	16	57	Not reached	3.1. Besides the optic trocar, another 2 trocars are needed (2 × 10 mm trocars)	28	21	51	Not reached			
					3.2. Besides the optic trocar, another 2 trocars are needed (1 × 5 mm and 1 × 10 mm trocars)	7	17	77	Agreement			
					3.3. Besides the optic trocar, another 2 × 10mm trocars and 2 × 5 mm trocars are needed	50	23	27	Not reached			
4. Two 5 mm trocars are required (one trocar is mandatory, the other optional)	2	18	80	Agreement								
5. A 30° laparoscopic camera is recommended	12	15	73	Not reached	5.1. A 30° laparoscopic camera is recommended	10	10	81	Agreement			
					5.2. A 0° laparoscopic camera is recommended	31	37	32	Not reached			
6. One 5 mm atraumatic grasping forceps is required	2	11	86	Agreement								
7. One Maryland grasper is required	7	13	80	Agreement								
8. Two needle holders are required	15	16	68	Not reached	8. At least one needle holder is recommended.	10	2	89	Agreement			
9. One pair of monopolar scissors is required	4	2	95	Agreement								
10. One bipolar forceps is required	2	7	92	Agreement								
11. One vessel sealing device can be used—optional	20	16	65	Not reached	11. One vessel sealing device can be used—optional	11	7	81	Not reached	6	94	Agreement
12. 4-0 polyglactin suture (on a 26 mm blunt or RB 1 needle) is required	7	28	64	Not reached	12.1. 4-0 polyglactin suture is recommended	4	18	79	Agreement			
					12.2. A 1/2 circle 26 mm needle is recommended	8	21	71	Agreement			
					12.3. A 5/8 circle 26 mm needle is recommended	24	41	37	Not reached	61	39	Not reached
13. 4-0 monofilament suture is required	4	15	81	Agreement								
14. One suction-irrigation device is required	0	7	93	Agreement								
15. One suction drain is required	14	19	68	Not reached								
					15.1. A drain is recommended	11	6	82	Not reached	28	72	Agreement
					15.2. A passive drain is recommended	11	20	69	Not reached	28	72	Agreement
					15.3. A suction drain is recommended	41	17	41	Not reached	89	11	Disagreement
16. A 4.8 or 6 French 24–28 cm double-J stent with a nitinol hydrophilic guidewire is required	2	0	98	Agreement								
*Pyeloplasty procedural steps*					*Pyeloplasty procedural steps*							
1. Cystoscopy and retrograde pyelography with double-J stent insertion is optional prior to laparoscopic pyeloplasty	23	23	53	Not reached	1. Cystoscopy and retrograde pyelography with stent insertion is optional prior to laparoscopic pyeloplasty	18	18	64	Not reached	21	79	Agreement
2. Three (to five) laparoscopic trocars are placed after obtaining pneumoperitoneum respecting triangulation principle	0	10	90	Agreement								
3. The periumbilical port (usually pararectal) is used for the insertion of the laparoscope in transperitoneal approach	0	10	90	Agreement								
4. Retroperitoneal approach needs balloon dilatation to create the working space for pyeloplasty	7	26	66	Not reached	4. Balloon dilation to create the working space for pyeloplasty is recommended for the retroperitoneal approach	2	20	78	Agreement			
5. A prestented ureter is easier to identify due to its rigidity, and presenting is recommended	36	30	34	Not reached	5. Stenting should be done before pyeloplasty	43	28	28	Not reached	89	11	Disagreement
6. A good exposure of the UPJ is recommended with cephalic dissection of the proximal ureter towards the pelvis	0	6	96	Agreement								
7. The ureter and the renal pelvis are identified and dissected from the surrounding tissues in order to (a) gain length for the anastomosis, (b) reduce its size, and (c) transpose it over crossing vessels	0	0	100	Agreement								
8. 4-0 polyglactin or monofilament stay suture is placed in the anterior portion of the upper ureter	10	16	73	Agreement								
*Dismembered pyeloplasty (Anderson-Hynes)*					*Dismembered pyeloplasty (Anderson-Hynes)*							
1. The ureter is spatulated on the lateral side: an incision is made in the ureter for 2–3 cm on craniocaudal (posterior posterolateral) border	2	8	90	Agreement								
2. The diseased UPJ is excised and redundant tissue is excluded; a small flap can remain until the end of the procedure for traction	2	3	95	Agreement								
3. The anastomosis should be tension free and knots should be outside the UPJ lumen	2	0	99	Agreement								
4. Suture with 3 mm bites of tissue, 3 mm apart for nonischaemic watertight anastomosis	0	12	88	Agreement								
5. When a two–running suture technique is used, start by creating the posterior wall of the anastomosis by running the first stitch outside in on the ureter and inside out on the pelvis	4	9	88	Agreement								
6. After having the double-J stent placed properly, the anterior part of the anastomosis is performed with the second stitch using the same principles	0	2	98	Agreement								
7. At the end of the suture line, the stitch should lie at the outside part of the pelvic wall and the two stitches (anterior and posterior) are sutured together	6	6	89	Agreement								
8. Place one stay suture at the spatulated ureter and one stay suture at the anterior part of the renal pelvis	5	20	75	Agreement								
9. The following steps are the same as for the running technique (posterior wall, double-J stent insertion, anterior wall), but with interrupted sutures	2	11	87	Agreement								
10. The anastomosis is finished alongside the ureteral spatulation length. A three-point suture (renal pelvis out in, ureter in out in, renal pelvis in out) can be used at the end	3	23	73	Agreement								
*Foley Y-V plasty*					*Foley Y-V plasty*							
1. Outline a widely based triangle of a V-shaped flap and placement of stay suture on its border, with the base of the V-shaped flap on the medial aspect of the renal pelvis and the tip of the V shape flap at the UPJ	7	21	72	Agreement								
2. Incision of the apex of the flap along the lateral border of the proximal ureter into the normally calibrated ureter	5	20	75	Agreement								
3. Placement of a double-J stent	3	7	90	Agreement								
4. Approximation of the apex of the flap to the inferior aspect of the ureterotomy incision	3	14	83	Agreement								
5. Approximation of the posterior walls with interrupted or running suture	7	12	81	Agreement								
6. Anastomosis of the anterior walls	5	12	83	Agreement								
7. A double-J stent is passed through one of the trocars following the initially placed nitinol hydrophilic guidewire and advanced into the ureter and urinary bladder	4	8	88	Agreement								
8. The correct placement of the double-J stent should be confirmed by fluoroscopy (laparoscopic surgery) or flexible cystoscopy, or by instilling a dye into the bladder	25	18	56	Not reached	8. The correct placement of the double-J stent should be confirmed by fluoroscopy (laparoscopic surgery) or flexible cystoscopy, or by instilling a dye into the bladder	25	15	60	Not reached	41	59	Not reached

UPJ = ureteropelvic junction.

About the equipment required, it was agreed that Maryland grasper, monopolar scissors, bipolar forceps, and a suction device are needed; at least one needle holder is recommended; and a balloon dilator is needed if retroperitoneal access is performed. Regarding the procedural steps, a good exposure of the ureteropelvic junction (UPJ) with cephalic dissection of the proximal ureter towards the pelvis is recommended, the diseased UPJ is removed, and redundant tissue is excluded. However, a small flap may remain until the end of the procedure for traction. The anastomosis should be tension free, and the knots should be outside the lumen of the UPJ. There was no consensus that the correct placement of the JJ stent should be confirmed by fluoroscopy, flexible cystoscopy, or instilling a dye into the bladder.

### 3.4 Consensus panel expert meeting

In the online consensus expert panel meeting, 33 statements were discussed to find “agreement” or “disagreement”. Nineteen experts participated in the meeting and voted; seven statements (21%) reached an agreement. Two statements, both in the pyeloplasty section, were in >70% disagreement: “a suction drain is recommended” and “stenting should be done before pyeloplasty”.

## 4 Discussion

This study is the first of its kind, focusing on developing an intermediate laparoscopic urological skills curriculum known as LUSs2. Our group utilised a unique approach based on CTAs to meticulously describe the necessary equipment, the step-by-step technique, and the resolution of potential complications that may arise in various urological procedures. This method breaks down complex surgical procedures into discrete cognitive components, revealing cognitive processes crucial for successful execution.

Once the curriculum was formulated and discussed among the group and invited collaborators, our focus shifted to ensuring its validity and effectiveness. We employed the highly rigorous Delphi consensus methodology, a systematic process involving a panel of esteemed European experts within the urological laparoscopy domain. Through no more than two rounds of discussion and an additional consensus meeting, the panel reached an agreement on nearly all curriculum statements. Those that were not agreed upon are a matter of the surgeon’s preference and do not impact this intermediate task's overall generalisability and degree of standardisation. This process allowed for a comprehensive evaluation of the curriculum’s content, structure, and relevance, while providing a platform for these experienced surgeons to contribute their expertise further and refine the curriculum.

The escalating complexity of surgeries and the integration of advanced technologies such as laparoscopy, endoscopy, and robotics underscore the urgent need for standardised training curricula. With limitations in trainee working hours, heightened expectations for operational results, and the imperative to reduce complications and hospital stays, the importance of effective training methods, including cadaveric, animal, and virtual simulations, is magnified. However, these methods are often costly and inaccessible, emphasising the necessity for a validated, standardised curriculum.

Data from a survey conducted by the European Society of Residents in Urology noted that only 44% of surveyed residents had a training centre for simulation in laparoscopy, and only 67% had participated in practical courses on laparoscopy [3]. Although these data could be influenced by the progressive availability of robotic surgery in some residency programmes, the lack of laparoscopic training facilities and courses is evident. The same study found a positive association between training course participation and confidence in performing surgeries [3]. Studies estimate that around 70% of vital steps can be missed when taught by experts in lectures [9]. This may result from automation when surgeons reach the expert level. This could significantly affect the teaching process as experts may lose their conscious awareness of certain parts of procedures. Moreover, being an expert surgeon or having mastered surgical techniques does not translate into being an expert teacher and having a vocation as an educator [45].

Given these challenges, we advocate for the initial integration of CTAs into all surgical learning programmes. Compared with other motor-based interventions, CTAs offer several benefits, including ease of administration, cost effectiveness, and significant training impact [9]. We recommend prioritising cognitive skills before psychomotor skills training, with CTAs and hands-on courses complementing operating room training.

While our study makes significant strides in developing and validating an intermediate laparoscopic urological skills curriculum, it has limitations, particularly our reliance on survey-based data. First, the response rate and the inherent selection of respondents can affect the generalisability of our findings. Additionally, the phrasing of statements and the range of response options can lead participants towards specific answers. Lastly, while surveys provide valuable insights into the perceptions and experiences of experts, these do not capture objective measures of competency improvement. Despite the limitations, the Delphi consensus process proved to be an effective validation mechanism, allowing for the synthesis of diverse expert opinions. Its anonymous nature prevented any dominant influence, ensuring a true consensus. The process was managed efficiently through e-mails, negating the need for physical meetings.

Future studies should incorporate objective, performance-based assessments to complement survey findings, providing a more rounded evaluation of the curriculum's impact on surgical proficiency. Looking forward, we are developing an examination and certification process for the LUSs2 curriculum. This initiative is a critical step towards formalising the competencies acquired through this innovative training framework, aligning with the broader goals of the EAU and the ESU to elevate the standards of urological surgical education and enhance patient care.

## 5 Conclusions

LUSs2 is the first development of a laparoscopic surgery curriculum in urology beyond the basic steps. The combined approach, uniting CTAs and Delphi consensus, not only facilitated the creation of a robust and finely tuned surgical training curriculum, but also enhanced the collaborative spirit that dives into medical education led by experts.

  ***Author contributions*:** Patricia J. Zondervan and Diego M. Carrion had full access to all the data in the study and take responsibility for the integrity of the data and accuracy of the analysis.

  *Study concept and design*: Zondervan, Rivas, Carrion, Veneziano, Somani.

*Acquisition of data*: Zondervan, de Oliveira, Rivas, de Vries, Bujoreanu, Brinkman, Silvestri, Carrion, Socarras.

*Analysis and interpretation of data*: Zondervan, Rivas, Carrion, Socarras, Baekelandt, Veneziano, Somani.

*Drafting of the manuscript*: Zondervan, Rivas, Carrion, Socarras, Baekelandt, Veneziano, Somani.

*Critical revision of the manuscript for important intellectual content*: Zondervan, de Oliveira, Pini, Rivas, de Vries, Bujoreanu, Brinkman, Silvestri, Carrion, Socarras, Baekelandt, Veneziano, Somani.

*Statistical analysis*: Zondervan, Rivas, Carrion, Socarras, Baekelandt, Veneziano, Somani.

*Obtaining funding*: Erasmus + grant.

*Administrative, technical, or material support*: Julie Landsman.

*Supervision*: Zondervan, Veneziano, Somani.

*Other*: Initial development of all CTAs: renal hilum dissection—Skolarikos, Petrut, Veneziano; major vessel injury—Veneziano, Skolarikos, Greco; enucleation and renorrhaphy—Veneziano, Skolarikos, Pini; VU anastomosis—Tourinho-Barbosa, Sanchez-Salas, Alvarez-Maestro; pyeloplasty—Skolarikos, Alvarez-Maestro, Zondervan.

  ***Financial disclosures*:** Patricia J. Zondervan certifies that all conflicts of interest, including specific financial interests and relationships and affiliations relevant to the subject matter or materials discussed in the manuscript (eg, employment/affiliation, grants or funding, consultancies, honoraria, stock ownership or options, expert testimony, royalties, or patents filed, received, or pending), are the following: None.

  ***Funding/Support and role of the sponsor***: This study was supported within the SISE project by the Erasmus + grant, project number 2020-1-NL01-KA203-064721 (https://sise-urology.com/).

  ***Acknowledgements*:** Delphi support: Julie Landsman.

## References

[1] Ritchie A., Pacilli M., Nataraja R.M. (2023). Simulation-based education in urology—an update. Ther Adv Urol.

[2] Brinkman W.M., Tjiam I.M., Schout B.M.A. (2014). Results of the European Basic Laparoscopic Urological Skills examination. Eur Urol.

[3] Carrion D.M., Rodriguez-Socarrás M.E., Mantica G. (2020). Current status of urology surgical training in Europe: an ESRU-ESU-ESUT collaborative study. World J Urol.

[4] Checcucci E., Puliatti S., Pecoraro A. (2024). ESRU-ESU-YAU_UROTECH survey on urology residents surgical training: are we ready for simulation and a standardized program?. Eur Urol Open Sci.

[5] de Oliveira T.R., Cleynenbreugel B.V., Pereira S. (2019). Laparoscopic training in urology residency programs: a systematic review. Curr Urol.

[6] Militello L.G., Hutton R.J. (1998). Applied cognitive task analysis (ACTA): a practitioner’s toolkit for understanding cognitive task demands. Ergonomics.

[7] Ahmad K., Bhattacharyya R., Gupte C. (2020). Using cognitive task analysis to train orthopaedic surgeons—is it time to think differently?. A systematic review. Ann Med Surg.

[8] Edwards T.C., Coombs A.W., Szyszka B., Logishetty K., Cobb J.P. (2021). Cognitive task analysis-based training in surgery: a meta-analysis. BJS Open.

[9] Wingfield L.R., Kulendran M., Chow A., Nehme J., Purkayastha S. (2015). Cognitive task analysis: bringing Olympic athlete style training to surgical education. Surg Innov.

[10] Avery K.N.L., Chalmers K.A., Brookes S.T. (2018). Development of a core outcome set for clinical effectiveness trials in esophageal cancer resection surgery. Ann Surg.

[11] MacLennan S., Williamson P.R., Bekema H. (2017). A core outcome set for localised prostate cancer effectiveness trials. BJU Int.

[12] van der Poel H.G., Wit E.M., Acar C. (2017). Sentinel node biopsy for prostate cancer: report from a consensus panel meeting. BJU Int.

[13] von Rundstedt F.-C., Scovell J.M., Agrawal S., Zaneveld J., Link R.E. (2017). Utility of patient-specific silicone renal models for planning and rehearsal of complex tumour resections prior to robot-assisted laparoscopic partial nephrectomy. BJU Int.

[14] Whitehurst S.V., Lockrow E.G., Lendvay T.S. (2015). Comparison of two simulation systems to support robotic-assisted surgical training: a pilot study (Swine model). J Minim Invasive Gynecol.

[15] Yoon R., Del Junco M., Kaplan A. (2015). Development of a novel iPad-based laparoscopic trainer and comparison with a standard laparoscopic trainer for basic laparoscopic skills testing. J Surg Educ.

[16] Lin C.-C., Huang S.-C., Lin H.-H., Huang W.J., Chen W.-S., Yang S.-H. (2018). Naked-eye box trainer and training box games have similar training effect as conventional video-based box trainer for novices: a randomized controlled trial. Am J Surg.

[17] Schoenthaler M., Schnell D., Wilhelm K. (2016). Stereoscopic (3D) versus monoscopic (2D) laparoscopy: comparative study of performance using advanced HD optical systems in a surgical simulator model. World J Urol.

[18] Timberlake M.D., Stefanidis D., Gardner A.K. (2018). Examining the impact of surgical coaching on trainee physiologic response and basic skill acquisition. Surg Endosc.

[19] Zhang H., Wang J., Liu C. (2023). Development of a continuously perfused ex vivo kidney training model for laparoscopic partial nephrectomy: validity and efficiency. Int J Surg.

[20] Choussein S., Srouji S.S., Farland L.V. (2018). Robotic assistance confers ambidexterity to laparoscopic surgeons. J Minim Invasive Gynecol.

[21] Cicione A., Autorino R., Laguna M.P. (2015). Three-dimensional technology facilitates surgical performance of novice laparoscopy surgeons: a quantitative assessment on a porcine kidney model. Urology.

[22] Oussi N., Forsberg E., Dahlberg M., Enochsson L. (2023). Tele-mentoring—a way to expand laparoscopic simulator training for medical students over large distances: a prospective randomized pilot study. BMC Med Educ.

[23] Thinggaard E., Bjerrum F., Strandbygaard J., Konge L., Gögenur I. (2019). A randomised clinical trial of take-home laparoscopic training. Dan Med J.

[24] Sloth S.B., Jensen R.D., Seyer-Hansen M., Christensen M.K., De Win G. (2022). Remote training in laparoscopy: a randomized trial comparing home-based self-regulated training to centralized instructor-regulated training. Surg Endosc.

[25] Paterson C., McLuckie S., Yew-Fung C., Tang B., Lang S., Nabi G. (2016). Videotaping of surgical procedures and outcomes following extraperitoneal laparoscopic radical prostatectomy for clinically localized prostate cancer. J Surg Oncol.

[26] Veneziano D., Morgia G., Castelli T. (2021). Evaluation of the “Teaching Guide for Basic Laparoscopic Skills” as a stand-alone educational tool for hands-on training sessions: a pilot study. World J Urol.

[27] Zattoni F., Morlacco A., Cattaneo F. (2017). Development of a surgical safety training program and checklist for conversion during robotic partial nephrectomies. Urology.

[28] Desroches B., Porter J., Bhayani S., Figenshau R., Liu P.-Y., Stifelman M. (2021). Comparison of the safety and efficacy of valveless and standard insufflation during robotic partial nephrectomy: a prospective, randomized, multi-institutional trial. Urology.

[29] Gheza F., Raimondi P., Solaini L. (2018). Impact of one-to-one tutoring on fundamentals of laparoscopic surgery (FLS) passing rate in a single center experience outside the United States: a randomized controlled trial. Surg Endosc.

[30] Goldin S.B., Horn G.T., Schnaus M.J. (2014). FLS skill acquisition: a comparison of blocked vs interleaved practice. J Surg Educ.

[31] Abaza R., Ferroni M.C. (2022). Randomized trial of ultralow vs standard pneumoperitoneum during robotic prostatectomy. J Urol.

[32] Kaulfuss J.C., Kluth L.A., Marks P. (2021). Long-term effects of mental training on manual and cognitive skills in surgical education—a prospective study. J Surg Educ.

[33] Uysal D., Gasch C., Behnisch R. (2021). Evaluation of new motorized articulating laparoscopic instruments by laparoscopic novices using a standardized laparoscopic skills curriculum. Surg Endosc.

[34] Wu Q.-F., Kong H., Xu Z.-Z., Li H.-J., Mu D.-L., Wang D.-X. (2021). Impact of goal-directed hemodynamic management on the incidence of acute kidney injury in patients undergoing partial nephrectomy: a pilot randomized controlled trial. BMC Anesthesiol.

[35] Bic A., Mazeaud C., Salleron J. (2023). Complications after partial nephrectomy: robotics overcomes open surgery and laparoscopy: the PMSI French national database. BMC Urol.

[36] Zaid H.B., Parker W.P., Lohse C.M. (2017). Patient factors associated with 30-day complications after partial nephrectomy: a contemporary update. Urol Oncol.

[37] Bove P., Bertolo R., Sandri M. (2019). Assessing the impact of renal artery clamping during laparoscopic partial nephrectomy (LPN) for small renal masses: the rationale and design of the CLamp vs Off Clamp Kidney during LPN (CLOCK II) randomised phase III trial. BJU Int.

[38] Lu Q., Zhao X., Zhang S. (2024). Robot-assisted simple enucleation versus standard robot-assisted partial nephrectomy for low- or intermediate-complexity, clinical T1 renal tumors: a randomized controlled noninferiority trial. Eur Urol Oncol.

[39] Badawy H., Zoaier A., Ghoneim T., Hanno A. (2015). Transperitoneal versus retroperitoneal laparoscopic pyeloplasty in children: randomized clinical trial. J Pediatr Urol.

[40] Buffi N.M., Lughezzani G., Fossati N. (2015). Robot-assisted, single-site, dismembered pyeloplasty for ureteropelvic junction obstruction with the new da Vinci platform: a stage 2a study. Eur Urol.

[41] Gatti J.M., Amstutz S.P., Bowlin P.R., Stephany H.A., Murphy J.P. (2017). Laparoscopic vs open pyeloplasty in children: results of a randomized, prospective, controlled trial. J Urol.

[42] Khoder W.Y., Waidelich R., Ghamdi A.M.A., Schulz T., Becker A., Stief C.G. (2018). A prospective randomised comparison between the transperitoneal and retroperitoneoscopic approaches for robotic-assisted pyeloplasty in a single surgeon, single centre study. J Robot Surg.

[43] Silay M.S., Danacioglu O., Ozel K., Karaman M.I., Caskurlu T. (2020). Laparoscopy versus robotic-assisted pyeloplasty in children: preliminary results of a pilot prospective randomized controlled trial. World J Urol.

[44] Huang J., Zhang J., Wang Y. (2016). Comparing zero ischemia laparoscopic radio frequency ablation assisted tumor enucleation and laparoscopic partial nephrectomy for clinical T1a renal tumor: a randomized clinical trial. J Urol.

[45] Anderson C.I., Gupta R.N., Larson J.R. (2013). Impact of objectively assessing surgeons’ teaching on effective perioperative instructional behaviors. JAMA Surg.

