# Supplementary material for: Development of the European Laparoscopic Intermediate Urological Skills LUSs2 Curriculum: A Delphi Consensus from the European School of Urology
Source: Eur Urol Open Sci. 2024 Sep 13;69:22–50. doi: 10.1016/j.euros.2024.08.023 (PMC11416681; doi:10.1016/j.euros.2024.08.023)
Supplement: Supplementary Data 1 [file mmc1.docx]

**Supplementary material**

*Cognitive Task Analysis for E-LUS, step 2*

Contents:

[CTA Renal hilum dissection 2](#_Toc175310854)

[CTA Laparoscopic (or robotic) Pyeloplasty 13](#_Toc175310855)

[CTA Kidney tumour enucleation and renorrhaphy 27](#_Toc175310856)

[CTA Laparoscopic MVI repair 44](#_Toc175310857)

[CTA Laparoscopic Vesicourethral anastomosis 52](#_Toc175310858)

[Supplementary Table 1. Delphi Round Participation 66](#_Toc175310859)

# CTA Renal hilum dissection

**A. Indications**

1. As a step in the radical nephrectomy
2. As a step in the partial nephrectomy
3. As a step in the retroperitoneal lymph node dissection

**B. Contraindication**

1. Limited experience of the surgical team.
2. Pathological conditions of the renal hilum such as:
   - - Enlargement of lymph nodes around the hilum
     - Large retroperitoneal masses
     - Extensive Fibrosis

**C. Equipment**

- 5mm atraumatic grasping forceps
- 5mm straight (Maryland) dissecting forceps
- 5mm right angled dissecting forceps
- 5mm bipolar dissecting forceps
- 5mm blood vessel sealing device
- Monopolar or bipolar scissors
- 5mm Endoclip applier and 5mm clips
- 10 mm Endoclip applier and 10 mm clips
- Vessel loops (two different colours) for artery and vein occlusion

-or-

- 2 long (10mm) and 2 two short (6mm) pieces of a silicone 10F catheter as tourniquete
- Suction – irrigation device (can be used for irrigation suction and tissue dissection)
- Bulldog clamp applier
- Bulldog 5 mm straight – Bulldog 5 mm curved – bulldog 10mm straight – Bulldog 10mm curved
- Satinsky clamp
- Crawford laparoscopic curved clamp
- Hem-o-lock clips XL (in case of radical nephrectomy)
- EndoGia stapler with a 45-60mm vascular cassette (in case of radical nephrectomy

**D. Procedure steps ^1^**

**Preparation**

Check images to identify anatomy, identify best approach to the hilum, number of arteries, veins, location, abnormalities.

**Approach**

1. *Transperitoneal*
2. *Retroperitoneal*

**1. Transperitoneal approach.** Renal hilum can be approached by following three ways:

- Ascendant (following ureter) (*preferable*)
- Descendant (following Morrison space or cleavage plane between spleen and upper pole)
- Direct (straight on the hilum)

Despite the pathway used, the isolation of its structures (renal vein and renal artery) is achieved by their dissection as follows:

*Renal hilum dissection for right/left kidney*

- Rise up the lower kidney pole with the non-dominant hand along with the ureter and gonadal vein (left side), to expose the psoas muscle and to allow an easier dissection of the fatty tissue.
- Apply a vertical dissection to the fatty tissue overlying the psoas muscle. Dissection has to be gentle in order to allow visualization of tiny vessels that might need to be sealed, to avoid unwanted bleeding. Search for tiny tubular structures that are not detaching under the gentle vertical dissection produced with the dominant hand. Due to the small diameter, they might also have a very light colour, as opposed to the usual bigger vascular structures.
- While dissecting the fatty tissue, the non-dominant hand is progressively repositioned, moving cranially. Ureter and gonadal vein are lifted up to allow an easier and faster identification of the pedicle.
- Cut connective tissue even with no cauterization.
- Use cauterization when cutting adhesions, after a wise dissection. Adhesions are usually typical for the absence of cleavage planes and might have a harder consistency when compared to regular connective tissue.
- To ensure controlling the hilum before it bifurcates proximally to the kidney, it can be useful to move medially and search for the cava/aorta.
- During vertical dissection of the peri-hilar fat, the renal vein is the first to show up. Search for a flat, purple surface with a tiny trophic vessel overlay. The renal vein might be also found on the left side by simply following the gonadal vein.
- When the renal vein is first seen, its surface has to be considered as the new cleavage plane to be followed in order to take apart the fatty tissue to achieve a full vascular exposure.
- A Maryland dissector can help in case of adhesions or tight/absent cleavage planes over the vessels. Apply its closed tip to the point that you want to dissect and open it firmly but slowly. In order to produce a good dissection, the tip will have to be as close as possible to the right cleavage plane.
- Once the medial of the renal vein is fully exposed, the posterior one can be freed with an angled dissector or (in case it’s not available) with the Maryland dissector. Any curved instrument might be beneficial for it. This manoeuvre has to be carried out very carefully, as the renal artery is usually placed right behind the vein.
- Meanwhile, the non-dominant hand continues to lift up the kidney, to allow a correct dissection and visualization of the structures.
- The renal artery is usually located posteriorly and cranially to the renal vein. Search for: tubular structure of a smaller diameter if compared to the vein, pulsating, with a light red color and tiny surrounding trophic vessels. Its pulsation is usually typical as it tends to slightly assume an S shape. Check for aberrant arteries or arterial branches when you see the artery or when you work closely to the kidney and not on the level of the cava or aorta.
- In order to facilitate the exposure of the artery, remember to apply an effective lifting of the kidney with the non-dominant hand. In left-sided transperitoneal nephrectomy, one can have difficulty identifying the renal artery because it is lying either right behind or slightly superior to the renal vein. In this situation, the gonadal vein is clipped and transected at some distance from the renal vein.
- Special care has to be taken during the dissection of the renal artery as the surrounding fat may contain small vessels which could be damaged by excessive tension. This could therefore lead to bleeding accidents which might be challenging to solve, given the tiny working spaces.
- In order to complete the dissection of the renal artery (posterior face), place an angled dissector between it and the vein and open it slowly on its back. While passing between the vessels, the branches of the dissector have to be rotated 90° to achieve a vertical position.
- The renal artery can be eventually marked by placing a vessel loop, when needed.
- At the beginning of the procedure vessel tourniquets should be prepared as follows^2^: two vessel loops, one red (artery, vessel loop and the 10mm silicone tube) and one blue (vein and the 6 mm silicone tube) are required. The loops are folded, passed through the tubes, and then passed through a 12 mm trocar. The looped end of the vessel loop is passed around the vessel (first the artery and then the vein), and back over the end of the tube (like attaching a luggage tag). The free ends of the vessel loops are then fixed with a large (purple) Hem-o-lock clip so that they cannot fall apart. The tourniquets are left loose at the beginning, remain in position, until the very end of the procedure, permitting vascular control at any time if necessary. After the mobilization and manipulation of the kidney into the optimal position for tumor excision and reconstruction, traction on the end of the vessel loop, with application of a second XL (gold) Hem-o-lock firmly against the end of the tube, causes vessel occlusion.
- The dissection can be considered completed when each vessel is freed enough to safely place 3 clips or a bulldog clamp or the tourniquets (in case of a partial nephrectomy under warm ischemia)
- Depending on the procedure (adrenal sparing or not) the vein might be clipped and cut above the adrenal branch (adrenal sparing) or below the adrenal branch (not adrenal sparing).

**2. Retroperitoneal approach^3,4^:** Renal hilum can be achieved by ascendant approach. Ascendant (following ureter).

The isolation of its structures (renal vein and renal artery) is achieved by their dissection as follows:

- Expand the Gaur balloon in the retroperitoneal space to create the operating field.
- Kidney lower pole is identified, still covered with the peri-and para-renal fat.
- Lift up the lower pole of the kidney along with the ureter to allow access to the hilum.
- Identification of the vena cava/aorta will help an easier find of the hilum. Search for: tubular purple medial structure (vena cava) or pulsating red tubular structure (aorta).
- In the retroperitoneal approach the renal artery is the first to be identified, as the pedicle is dissected from the posterior side.
- In many retroperitoneoscopic cases the artery is obvious once the kidney is lifted up, but in others—usually obese men—the landmarks are indistinct and the location of the artery cannot be determined in the usual fashion (looking for pulsations). The artery though is almost always right in front of the port at the base of the 12th rib, or at most a centimeter or two cephalad to this. This is an amazingly consistent relationship, and if you are lost it can help you get started every single time.
- As described before, applying traction by lifting the lower kidney pole is critical to allow an easier dissection of the hilum, even in the retroperitoneal approach. At this point, it is important to keep the dissection in the flimsy white fibro-areolar tissue along the ipsilateral great vessel. One must stay anterior to the ipsilateral great vessel, taking care not to stay posterior to it. If dissection is proceeding in the yellow peri-renal fat, one is probably dissecting too close to the renal parenchyma. Again, good lateral counter-traction is important to place the renal hilum on stretch. In general, the renal hilum is located at an angle of 45 to 60 degrees from the vertical. This is the angle that the shafts of your instruments outside the patient’s body will be describing when you find the renal vessels.
- The renal artery is posterior, and the renal vein is anterior and usually caudal (inferior) to the renal artery. Before beginning dissection on the renal artery or vein, the horizontal positions of the major vessels (aorta on the left side, vena cava on the right: both parallel to the psoas) and vertical pulsations of the fat-covered renal artery laterally are looked for, and almost always visualized. One must remember that during renal retroperitoneoscopy the psoas is the constant anatomic landmark: the psoas “is your best friend.”
- Also in this case, the dissection is complete when each vessel is freed enough to safely place 3 clips, a bulldog clamp or a tourniquet (in case of a partial nephrectomy under warm ischemia)

**E. “Do’s and Don’ts”**

**Surgeon**

- **“Do’s”**
- Evaluate the hilar vasculature as well as possible using preoperative imaging. It is helpful to know if there is more than one renal artery or vein. In addition, on occasion, one could identify large lumbar veins emanating from the left renal vein. Anomalies such as retro-aortic renal vein, a branch of the right renal artery traveling anterior to the inferior vena cava, as well as a left inferior vena cava can be encountered.
- Be careful, apply slow and precise manoeuvers.
- Don’t move instruments outside the field of view if you’re not sure of their location or feel resistance.
- Always apply traction to widen your working area and highlight eventual vascular structures
- Search for cleavage planes and avascular lines to make your way to the main vessels.
- Aim to isolate the major veins and arteries in order to get full control, in case of eventual bleedings.
- In case of tight adhesions, proceed with small bites and try to dissect small areas.
- Prefer blunt-tip instruments
- Prepare the tourniquets on the nurse table
- Check the adequacy and the integrity of the instruments
- **“Don’ts”**
- Don’t cut before having completely exposed the inner structures
- Don’t advance instruments into small holes before having seen what’s hidden behind (may cause inadvertent injury of a vessel, which might be challenging to control)

**Assistant**

- **“Do’s”**
- Hold the camera steady
- Keep the working field in the center of the camera view
- Be careful, apply slow and precise maneuvers.
- Ask the surgeon beforehand if he wants any “specific” movements or turns of the camera when dissecting the hilum
- Evacuate the smoke often by leaving temporarily the gas valve of a 5 mm trocar open
- **Don’ts**
- Don’t move instruments outside the field of view if you’re not sure of their location or feel resistance.
- Do not advance the camera in close proximity to the field to avoid getting dirty from blood or smoke

**F: How to handle problems**

- In case of unexpected, sudden bleeding, clean the area with suction device and try to understand first of all where the bleeding is coming from. Applying pressure for 5-10minutes with a gauze pad, may seal a venous leak, gives time to think and prepare yourself on how the bleeding will be controlled. If the bleeding is minimal, after the above measures have been taken, many times you can leave this area to work in another area and the bleeding will have stopped in the interim.
- In case the bleeding comes from a small tear of the vena cava applying pressure and an haemostatic agent could solve the problem. In case of a small venotomy, just pinching the vena cava in that area and placing a row of titanium clips can secure haemostasis. Alternatively, the venous injury can be over-sewn with laparoscopic free- hand suturing. One can place a Hem-o-Lock clip or Lapra-Ty clip on the end of the suture instead of a knot and then use that to run a figure-8 stitch to close the vena cava. Before exiting, the site should be inspected after 5 to ten minutes of disinflation.
- In case the bleeding comes from the gonadal vein, try to isolate it caudally in a different area until you achieve room enough to place a clip. This will slow down immediately the bleeding cranially.
- In case the bleeding comes from a non-identifiable tiny vessel, apply bipolar cauterization while cleaning with the suction device. If the bleeding is overriding the suction, help yourself with increasing the pneumoperitoneum to 20mmHg and increase the gas flow to high level (40 Litre/minute) (especially in the presence of aggressive suctioning). Introducing a gauze through a trocar and applying local compression might help to stop the bleeding.
- In case the bleeding is coming from a branch of the renal vein/artery, try to grasp the area with a blunt dissector or grasper to stop the bleeding and then proceed to dissection of the surrounding tissue, until there is room enough to apply a clip (titanium of small hem-o-lock). Repair renal vessel injury intra-corporeally using 5-0 Prolene suture in a figure of 8 fashion.
- When significant bleeding is encountered from a renal hilar injury, an effort should be made to repair it. However, when repair is not possible or the bleeding is too brisk and the vein and artery are satisfactorily dissected, then you can use an Endo-GIA stapler temporarily to occlude the renal hilum en-bloc. If bleeding is controlled, an attempt at repair should be made. If repair is unsuccessful or not feasible and care has been taken not to staple inadvertently across the aorta, inferior vena cava, adrenal gland or the pancreas, the hilum is then divided en-bloc.
- In case the kidney is falling down and compressing the hilum during a bleeding, consider the positioning of an additional port to help keeping on traction, while the both first-operator instruments work on the resolution of the complication.
- In case of an (laparoscopic) uncontrollable bleeding a prompt decision to convert to open surgery should be made. Iodine and sterile cover should already be anticipated on a possible conversion, patient is preferably moved to supine position and a midline or subcostal (chevron) incision should be made for adequate exposure of major vessels depending on surgeon's preference.

**TASK idea:**

Model: An apparatus is connected to a lap training box, including an horizontal plate and a moving rod articulated to one end of the plate. At the middle of their length, attachments are positioned (up and down) to hold a replica of a renal hilum, including one vein and one artery displaced in an anatomically correct position (easily identifiable one from each other). Each vessel will be pre-filled with fluid to assess eventual accidental lesions and will be provided with markers 2.5 cm distant one from each other. The two vessels will be surrounded by dissectable synthetic materials and arranged in a disposable pack.

Description: The trainee gets ready with 5mm straight (Maryland) dissecting forceps, 5mm right angled dissecting forceps, Monopolar or bipolar scissors, 5mm Endoclip applier and 5mm clips. The trainee raises up the moving rod with one instrument and starts dissecting the disposable pack, until the artery is properly isolated (the area over or below the markers is pepped enough to place the first clip). After the correct placement of the first clip, the trainee will decide to either continue by placing the other two clips on the artery or starting to dissect vein and proceed with clipping. All clips need to be placed proximally to the proximal marker (one clip) and distal to the distal marker (two clips). After clip placement has been completed, the trainee will cut the vessel in the area between the two markers.

Target time: to be defined

Time start/stop: The candidate touches the model/the trainee cuts the second vessel

**Errors:**

- Clips don’t enclose the whole vessel (assessed by fluid leakage by the remaining portion of the vessel)

- Clips intersect one of the markers

- Fluid coming out of one vessel before the clips are placed (accidental injury)

- Clip is applied on the vein first

- The vessel is not isolated for the whole length determined by the markers

**Possible case scenarios:**

- The candidate does never apply traction along the whole task: task failed

**G. References**

1. Surgery in motion School platform of the EAU
2. Janetschek G, Zimmermann R. Laparoscopic Transperitoneal Partial nephrectomy. In Stolzenburg JU, Turk IA, Liatsikos EN Laparoscopic and Robot-assisted surgery in Urology. Atlas of Standard Procedures Springer Editions
3. Gaur DD Retroperitoneal Laparoscopic Urology Oxford University press
4. Kumar U, Gill IS Tips and Tricks in Laparoscopic Urology Springer Edition
5. <https://bjui-journals.onlinelibrary.wiley.com/doi/epdf/10.1111/j.1464-410X.2011.10314.x>

# CTA Laparoscopic (or robotic) Pyeloplasty

1. **Indications**

- Symptomatic UPJ obstruction (flank pain)
- Urinary tract infection / stones
- Progressive renal deterioration
- Causal hypertension (rare)
- Impaired renal function in a solitary kidney or bilateral disease

1. **Relative contraindications**

- Previous surgery
- Renal inflammation

1. **Equipment**

*Laparoscopic Pyeloplasty*

Equipment depends on the surgeon and the technique (e.g standard, mini laparoscopy)

- One balloon dilator (for retroperitoneal access)
- Two 10mm trocars
- Two 5 mm trocar (one trocar is mandatory, the other optional)
- A 30-degree laparoscopic camera
- One 5 mm atraumatic grasping forceps
- One Maryland grasper
- Two needle holders
- One monopolar scissor
- One bipolar forceps
- One Harmonic scalpel (optional)
- 4x 3-0 / 2x 4-0 polyglactin / PDS suture (on a RB 1 needle) / 1x 2-0 vicryl
- One suction-irrigation device
- One suction drain
- 4.8 or 6 French 26-28 cm double–J stent with a nitinol hydrophilic guidewire

**D. Procedural steps**

Preliminary Access

*Transperitoneal Laparoscopic Approach*

- Cystoscopy and retrograde pyelography (with or without stent insertion) - optional
- Veress or Hasson techniques for peritoneal access - optional
- Three (to five) laparoscopic trocars are placed after obtaining pneumoperitoneum respecting triangulation principle
- The umbilical port is used for insertion of the laparoscope
- Colonic mobilisation for exposure of retroperitoneal structures is performed
- Mobilisation of the ipsilateral proximal ureter, UPJ and renal pelvis
- Pyeloplasty

*Retroperitoneal Laparoscopic approach*

- Cystoscopy and retrograde pyelography (with or without stent insertion) - optional
- Initial incision at the retroperitoneal space
- Balloon dilation of the retroperitoneal space
- CO_2_ retroperitoneum
- Next steps identical to the transperitoneal approach

Pyeloplasty

**Dismembered Pyeloplasty (Anderson-Hynes)**


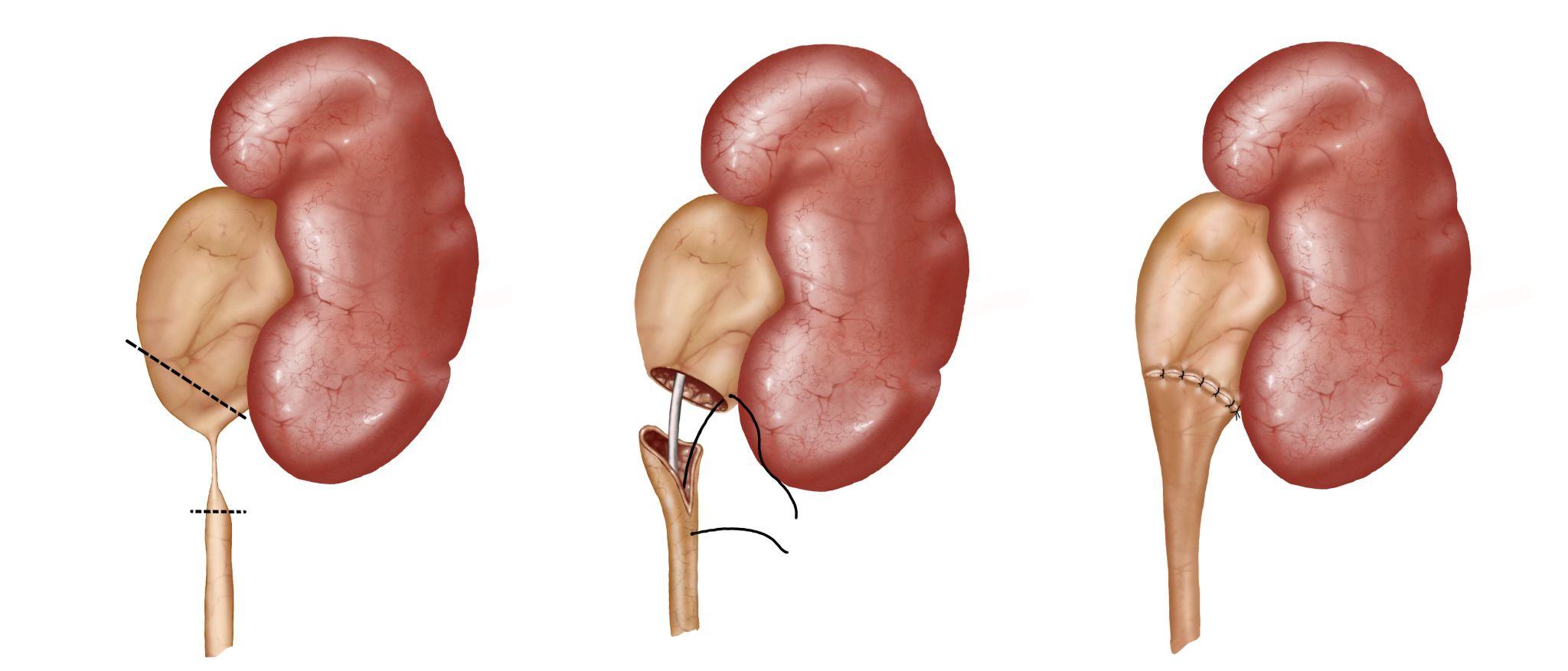


● *Identification of the proximal ureter in the retroperitoneum*

1. In the transperitoneal approach, after mobilisation of the colon and dissection of the retroperitoneal space, the first anatomical reference is the psoas muscle. Anterior to the psoas muscle are the ureter and the gonadal vein. The ureter appears as a soft tubular structure, slightly white coloured with surrounding trophic vessels and it is revealed by its peristalsis when touched.
2. In the retroperitoneal approach, after dissecting the retroperitoneum and the Gerota fascia, the first identified structure is the ureter. The dilated renal pelvis is the best landmark.
3. In difficult cases (e.g. previous surgery) or when the renal pelvis is pre-stented, it is best to first identify the psoas muscle or the lower pole of the kidney. The ureter lies on and below these two structures, respectively. A pre-stented ureter is easier to identify (when possible to place a stent) as its deformation is highly determined by the rigidity of the stent itself.
4. Moving over a well-exposed psoas muscle will allow an easy rise up of both ureter and gonadal vein. This manoeuvre will allow an easy path to the pelvis.

● *Cephalic dissection of the proximal ureter towards the renal pelvis*

1. You may gently grasp the ureter for retraction or lift the ureter up with the instrument held in the non-dominant hand, while dissecting the surrounding tissue with the instrument held in the dominant hand.
2. Care should be taken not to devascularize the ureter on a long segment.
3. Think ahead for the length of the ureter needed for anastomosis and the surgical technique in case of crossing vessels.

● *Mobilisation of the renal pelvis*

1. The renal pelvis should be fully dissected from the surrounding tissues and mobilised in order to a. gain length for the anastomosis b. reduce its size and c. transpose it over crossing vessels.
2. Retraction of the ureter and renal pelvis is required during this step. Instead of using a grasper and consequently needing a separate port, one way of retracting the ureter is to use a Keith needle passed through the abdominal wall under endoscopic vision hooking below the ureter and brought out to the tegument. The suture may then be placed on tension as necessary to elevate the ureter.
3. Once the UPJ has been exposed, fine 3-0 or 4-0 polyglactin stay sutures are placed in the anterior portion of the upper ureter and the anterior portion of the renal pelvis and both structures are gently mobilised. *This manoeuvre is optional, depending on the experience of the surgeon.*
4. The ureter and the UPJ area should be completely dissected free from the crossing vessels both cephalad and caudal to the vessel.

● *Transection of the UPJ*

1. The upper ureter is transected above the traction suture obliquely and anterior to posterior, leaving the medial blood supply undisturbed.
2. The renal pelvis and obstructed UPJ are transected similarly, leaving the traction suture in place at the most dependent portion of the inferior renal pelvis.
3. The ureter is incised for 2 to 3 cm cranio-caudal (posterior-posterolateral border), to provide an open tube for triangulated anastomoses to be made at the end.
4. At the same time the renal pelvis is trimmed to exclude redundant tissue. Reduce the renal pelvis with the UPJ not dismembered, so that the pelvis is completely stabilised. Incise at the point the most proximal incision will be, excise a portion of the renal pelvis and then remove it.
5. The diseased UPJ is excised, although a small flap can remain connected to the ureter as a handle, reducing unnecessary contact and traction with the area to be anastomosed. This flap is excised towards the end of the procedure.
6. With the UPJ dismembered, grasp the ureter (or the remaining UPJ portion) on the most lateral aspect, and pull it toward you and down. The ureter is held in a straight line so that, especially in laparoscopic surgery, the camera looks down to the opening of the ureter.
7. Next, one blade of the scissors is inserted into the open ureter, the blades are closed and the ureter is spatulated over 1 cm. Repeat the same cut for 1 cm more spatulation.
8. For robotic approach: Using the maximum angulation offered by the EndoWrist round-tip scissors, the instrument works like a Potts scissors. This allows excellent control of the degree and length of spatulation.

● *Anastomosis (Principles)*

1. *The anastomosis should be tension-free and knots outside the UPJ lumen.*
2. The anastomosis is always performed from the apex of the ureteral spatulation toward the pelvis with the initial stitch placed at the apex.
3. Suture lines run from a lateral to medial direction to allow full overview of the anastomosis from beginning to the end.
4. Suture with 3 mm bites of tissue, 3 mm apart for non-ischemic watertight anastomosis.
5. Either interrupted or two separate running sutures (20-cm 4-0 polyglactin suture) along the anterior and posterior lines of the anastomosis are used.
6. For the anastomosis use a 4-0 monofilament suture such as Monocryl®, 20cm in length, on an 26-mm blunt or RB-1 needle.
7. Alternatively, 4-0 polyglactin or PDS or braided sutures can be used.
8. Most surgeons start with the posterior wall, move to stent insertion, close the anterior wall and then the defect of the renal pelvis.
9. The order could be different depending on the surgeons’ preference and experience (anterior wall, renal defect, posterior wall).

● *Anastomosis (Running Technique)*

1. When a two running suture technique is used, starting by creating the posterior wall of the anastomosis, one should place one or two sutures at the heel of the ureter before doing anything else. If you are going to have a urine leak from a pyeloplasty this is where it is going to be. For these stitches you might also use a braided 4-0 suture, which will be easier to be tightened, thus allowing a secure closure of the apex before starting with the running suture.
2. Start from the ureter outside-in at the posterior part of the heel of the ureter and move to the renal pelvis form inside-out, then intracorporeally tie the suture ensuring the knot is on the outside.
3. Then place the other running stitch to the ureter from outside in at the anterior part of the heel of the ureter and move to the anterior part of the renal pelvis from inside out. Tie the knot on the outside. The later step can be done after the completion of the posterior wall of the anastomosis, but when it is initially performed it strengthens and stabilises the rest of the anastomosis.
4. Start the posterior part of the anastomosis by running the first stitch over and over from lateral to medial and outside in and inside out on the ureter to the pelvis, respectively.
5. At the end of the suture line the tie should lie outside of the renal pelvic wall. Remember to incorporate in the suture line also the medial aspect of the wide proximal edge of the ureter creating a “boat keel”. This suture will be tied with the one from the anterior anastomosis.
6. After having the double J stent properly placed, the anterior part of the anastomosis is performed.
7. Use the second stitch, start again at the heel of the ureter by running over and over from lateral to medial and outside-in in the ureter and inside-out in the pelvis.
8. At the end of the suture line the stitch should lie at the outside part of the pelvic wall.
9. Tie the two stitches (anterior and posterior) together.
10. You may want to keep the needles in place in case you need to suture any redundant tissue of the anastomosis to the pelvic wall, as the latter is sutured.
11. The pyelotomy superior to the pelvi-ureteric anastomosis is now closed. The renal pelvis is closed with a running suture starting from the superior aspect of the defect. When the running suture reaches the anastomosis it is tied on itself or with one of the previous stitches with or without incorporating a “bite” of the ureteral wall at the edge of the “boat keel”.

● *Anastomosis (Interrupted sutures)*

1. Place one stay suture at the heel of the ureter.
2. Place one stay suture at the anterior part of the renal pelvis.
3. Start the interrupted sutures (spaced 3 mm apart) from the posterior part of the ureteral corner by inserting the needle from outside in the ureter.
4. Insert the needle at the posterior part of the renal pelvis in an inside out fashion.
5. Make a knot outside the lumen and cut the suture, leaving a short length for retraction if needed.
6. Continue with interrupted sutures alongside the posterior wall of the anastomosis.
7. Insert a double j-stent.
8. Continue with interrupted sutures and create the anterior part of the anastomosis.
9. The anterior part is easier. With the dominant hand place the needle from outside into the pelvis side and from inside out to the ureter side.
10. Finish with interrupted sutures the anastomosis alongside the ureteral spatulation length.
11. If the renal pelvis was reduced, the defect is sutured with either interrupted or running sutures.
12. Take care to safely suture the junction between the anterior part of the ureter and the renal pelvis.
13. To close the triangular gap between the ureter and the renal pelvis, make a three-point suture (renal pelvis out-in, ureter in-out-in, renal pelvis in-out).

**Foley Y-V Plasty**


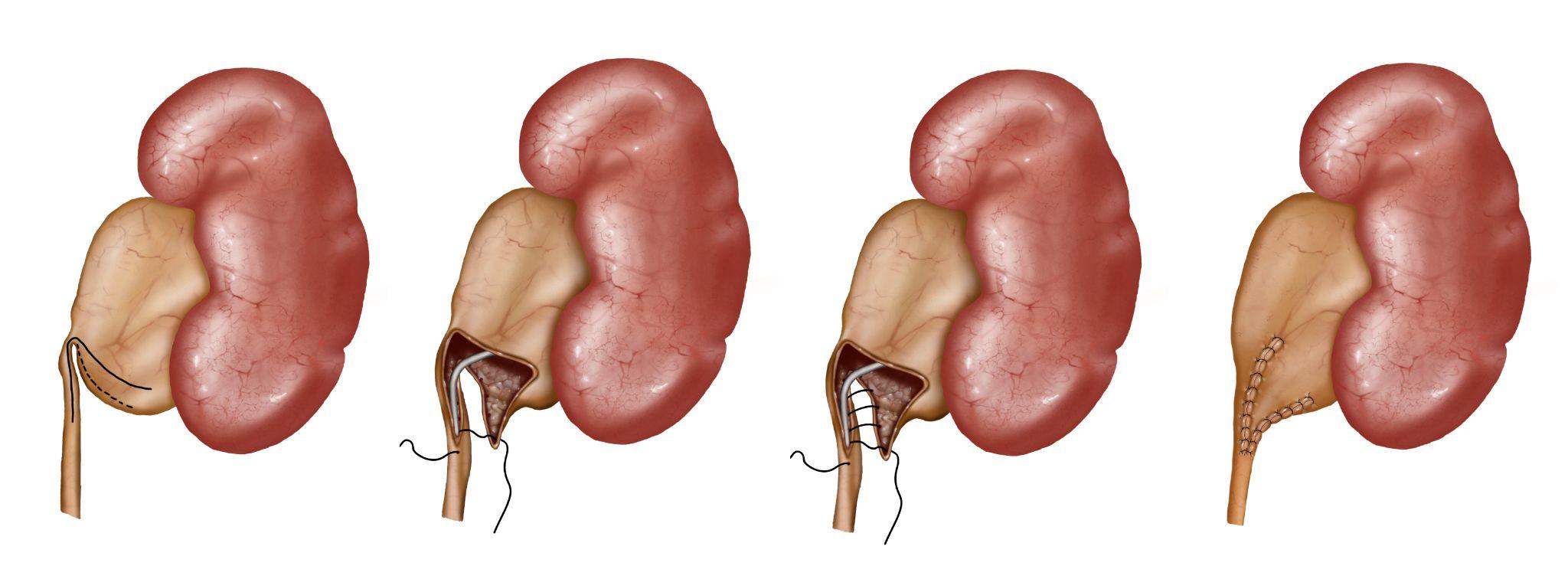


- Exposure of the renal pelvis and proximal ureter
- Outline a widely based triangular of V shaped flap and placement of stay suture on its border
- The base of the V shaped flap is positioned on the medial aspect of the renal pelvis while the apex at the UPJ
- Incision of the apex of the flap along the lateral border of the proximal ureter several millimetres into the normal calibre ureter
- Creation of the renal pelvic flap and excision of the ureter with scissors
- Placement of a double J stent
- Approximation of the apex of the flap to the inferior aspect of the ureterotomy incision with monofilament absorbable suture (polyglactin 4-0)
- Approximation of the posterior walls with interrupted or running suture (polyglactin 4-0)
- Anastomosis of the anterior walls

*Insertion of double J stent*

After the completion of the posterior wall of the anastomosis the double J stent should be placed. There are several ways to achieve this task.

1. A pre-stented ureter is easier to manipulate during this step.
2. An antegrade approach could be used. With the use of a spinal needle the anterior body wall is punctured in a line straight to the anastomosis. The needle is removed and a nitinol hydrophilic guidewire is inserted through the sheath and is manipulated through the anastomosis down to the ureter and the bladder.
3. Alternatively, a ureteric catheter is passed through one of the trocars and the guidewire is manipulated and advanced into the ureter and the bladder through the catheter.
4. Over the guidewire the double J stent is advanced into the bladder.
5. The type of the stent used depends on the surgeon's preference. Most commonly a 4.8-6Fr, 26-28 cm is used. The correct placement of the stent relies on the surgeon's experience but it can be confirmed by fluoroscopy (laparoscopic surgery), flexible cystoscopy, or by instilling 200cc of saline with indigo carmine into the bladder. As soon as leakage of the blue dye from the stent is noted at the anastomosis, the position of the distal coil of the stent into the bladder is confirmed.

**E. “Do’s and Don'ts”**

**Surgeon**

**Do’s**

- Do a wide spatulation of the ureter and renal pelvis.
- Perform spatulation on the lateral side (and not on the medial side, as the vascularisation is at the medial side).
- Do a precise mucosa-to-mucosa anastomosis.
- Do a tension-free anastomosis.
- Excise the faulty - stenotic UPJ segment.
- Perform adequate pelviolysis, especially when crossing vessels exist.
- Perform a transposition of possible “crossing vessels” according to Anderson Hynes pyeloplasty.
- Reduce the excessive renal pelvis (when needed) in a straight line with the anastomosis.
- Get facile with suturing with both hands. You may find it easier to suture with the right hand for right-sided pyeloplasty and with the left hand for left-sided pyeloplasty.
- Work with an experienced bedside laparoscopic surgeon.

**Don'ts**

- Do not pull the stitches excessively while suturing, to avoid dismantling the anastomosis.
- Do not leave excessive lengths of the stay sutures or the cut edges of the running sutures to avoid intermingling with the anastomotic line.
- Do not twist the anastomosis. The most common cause of torsion of the anastomoses is the wrong way of cutting the UPJ, spatulating the ureter or putting the first stitches.
- Do not cut the preplaced stent when dismembering the UPJ. When the stent is inadvertently cut, both pieces of the damaged stent should be removed.
- Do not leave a preplaced stent for a long time (7 to 10 days) prior to laparoscopic / robotic pyeloplasty. Edema will develop and the passes of the stitches will become difficult.
- Do not leave the crossing vessels lying directly across the reconstructing UPJ.
- Do not hesitate to put an extra trocar. A subxiphoid location is a straight shot to the UPJ.
- Do not place the robotic ports very close to each other. To avoid robotic arm collisions, the instrument arms must be 8-10 cm from the camera port (one fist distance).

**F: How to handle problems**

- In case there is leakage of the anastomosis an extra suture can be placed, with attentive care to not make the plasty ischemic due to too close suturing.
- In case there is tension on the anastomosis observed while suturing, a lateral dissection of the ureter (approx. 2cm - as minimal as possible) can be made to allow it to become tension free, but with attentive care to minimise ureteral devascularization during dissection. To avoid this, the proper plasty technique should be chosen, approximating the defect and tissue tension beforehand or it should be adjusted during suturing to obtain the best results. The tension on the anastomosis can be due to a bigger defect but also due to manipulation during suturing, associating tissue trituration. For this, a continuous running suture bringing the ureter to the pelvis step by step could be a good option.
- In case there is a crossing vessel over the anastomosis transposition is an option, even though dismembered technique is advisable.

**TASK idea:**

*Model:* synthetic replica of a Uretero-Pelvic Junction including pelvis, a 5mm stenotic segment and a 10cm-long ureter. The model needs to be water friendly and have a low friction to allow proper insertion of regular ureteric stents. The model needs to allow proper measurement of water tightness after the completion of the pyeloplasty. Materials used need to allow proper stitching without tearing (under average tension).

*Description:* The tutor mounts the model inside the box with the fixing system provided. The trainee gets ready with two needle holders, Maryland dissector and laparoscopic scissors. Sutures available: 2-0 braided and 3-0 to 4-0 monofilament. 4-0 braided suture is recommended.

One DJ stent is provided with guidewire and pusher: 4.8 or 6 French 26-28 cm double–J stent with a nitinol hydrophilic guidewire. The trainee performs the pyeloplasty in accordance with his technique of preference, after cutting away the stenotic segment. Prior to the completion of the plasty he will put in place the provided DJ stent. Reduction of the pelvis will be upon his preference.

After the completion of the task, the tutor will check water tightness of the anastomosis by flushing water in the model with a pressure of no more than 10cm of water (3LT perfusion bag is placed 10 cm higher than the model). Water-tightness test will be considered successful if 80% of the flushed water is collected from the end of the ureter.

*Target time:* TBD (Time limit is defined just for skill measurement and examination organisation purposes)

*Time start/stop:* The model is touched for the first time/the trainee declares the pyeloplasty as completed.

**Errors:**

- Water leakage over 20% of the total amount of flushed fluid
- Missing to put in place the DJ stent
- Occlusion of the ureter along the anastomosis
- Pelvis and ureter not properly aligned (twisted)
- Spatulation on the medial side

**Possible case scenarios:**

- The trainee cuts too much tissue, thus making the realignment difficult or impossible: task is considered as failed.
- The trainee applies excessive force to the ureter, thus detaching the model from its base: task is considered as failed.

**References**

1. Surgery in motion School platform of the EAU

2. Kreutzer N, Abulsorour S, Casey R, Stolzzenburg JU, Trub MC. Laparoscopic pyeloplasty. In Stolzenburg JU, Turk IA, Liatsikos EN Laparoscopic and Robot-assisted surgery in Urology. Atlas of Standard Procedures Springer Editions

3. Beerlage HP, Casey R, Kallidonis P, Liatsikos E, Stolzenburg JU Robot-assisted pyeloplasty. In Stolzenburg JU, Turk IA, Liatsikos EN Laparoscopic and Robot-assisted surgery in Urology. Atlas of Standard Procedures Springer Editions

4. Gaur DD Retroperitoneal Laparoscopic Urology Oxford University press

5. Kumar U, Gill IS Tips and Tricks in Laparoscopic Urology Springer Edition

# CTA Kidney tumour enucleation and renorrhaphy

**A. Indications^1^**

Nephron-sparing surgery (NSS) when compared to radical nephrectomy (RN):

1. has demonstrated a comparable non-inferiority CSS in organ-confined renal cell carcinoma (RCC) of limited size, respectively T-stage (pT1 <7cm);
2. same conclusion for larger tumors (clinical stage T2), even if a higher risk of perioperative complications should be considered;
3. has demonstrated to better preserve general kidney function, thereby lowering the risk of development of metabolic or cardiovascular disorders.

In view of the above, the risks and the benefits of PN should be discussed with patients with kidney tumours. Generally, PN should be considered, if technically feasible, in patients with a solitary kidney, bilateral renal tumours or CKD with sufficient parenchymal volume preserved to allow sufficient post-operative renal function^2^.

**B. Contraindications (relevant)**

Nowadays based on new advanced technology and different years of experience in that surgery the number of PN that could be performed increase exponentially. Anyway, treatment decision should be carefully individualized balancing comorbidities and frailty index of patients with the risks and benefits of NSS versus radical surgeries.

Partial nephrectomy (PN) is unsuitable in some patients with localized RCC due to:

- Insufficient volume of remaining parenchyma to maintain proper organ function;
- Renal vein thrombosis;
- Unfavourable tumour location e.g. adherence to the renal vessels or complete endophytic tumour;
- Use of anticoagulants (relative contraindications).

**C. Equipment**

- 2 x 10-12-mm trocar
- 3-5 x 5-mm trocar
- 1 x Laparoscopic Bipolar grasper
- 1 x Laparoscopic aspiration-irrigation device
- 1 x Laparoscopic monopolar scissors
- 1 x Laparoscopic right-angle dissector
- 1 x Laparoscopic Maryland forceps
- 1 x Laparoscopic 10-mm Satinsky or bulldog clamp applier
- 2 x Laparoscopic Needle holders
- 1 x Laparoscopic probe for intraoperative ultrasound (i.e. BK Medical, Helev, Denmark)
- 2 x Bulldog clamp (Scanlan® Reliance, St Paul, Usa; Aesculap®, B Braun, Germany)/ Tourniquet
- Optional:
  - Advanced sealing system can be helpful (i.e. Ligasure®, Covidien-Medtronic, Dublin, Ireland; Harmonic scalpel® (HS), Enseal®, Ethicon, Raritan, Usa; Vojant®, Applied Medical, California, Usa)
  - Air-seal insufflation management system offers capabilities in providing a stable pneumoperitoneum, constant smoke evacuation, and valve-free access.
- Sutures
  - Sliding clip renorrhaphy
    - CT-1, 36, 1/2C, Taperpoint, 0, polyglactin 20-25cm, preloaded with non-absorbable (Hem-o-Lock®, Weck-Teleflex, Wayne, Usa; Click'A-V®, Grena, Nottingham, UK) or absorbable (Lapra-Ty®, Ethicon, Raritan, Usa) clips
    - CT-2, 26, 1/2C, Taperpoint, polyglactin, 2/0, 20cm, preloaded with non-absorbable (Hem-o-Lock®, Weck-Teleflex, Wayne, Usa; Click'A-V®, Grena, Nottingham, UK) or absorbable (Lapra-Ty®, Ethicon, Raritan, Usa) clips
  - Sliding clip renorrhaphy with barbed suture
    - V-Lok V-30 30mm ½C, Taperpoint 2/0 23cm
- Non-absorbable (Hem-o-Lock®, Weck-Teleflex, Wayne, Usa; Click'A-V®, Grena, Nottingham, UK) or absorbable (Lapra-Ty®, Ethicon, Raritan, Usa) clips and Clip applier
- Haemostatic agents (e.g. Floseal® or TachoSil®, Baxter International, Illinois, Usa; Surgicel® Ethicon, Raritan, Usa)

**D. Procedure steps (list all techniques)**

- Preoperative planning based on imaging: dimension, shape, exophytic / endophytic, distance from calyces.
- Approach:
  - Anatomical access
    - Retroperitoneal approach
    - Transperitoneal approach
  - Ischemia
    - No (zero) ischemia
    - Warm
    - Selective/ super-selective clamping (with or without fluorescence)
    - Early arterial unclamping^3^

**1. Tumour exposition**

- Dissection of the renal pedicle; encircled renal artery with elastic vessel loops for retraction and *get ready* to warm ischemia with Satinsky/bulldog clamp/tourniquet
- Make a first incision on the perirenal fat, up to the kidney capsule visualization.
- Follow the cleavage plane between capsule and fat until the border of the tumour/adipose tissue covering the tumour (if the tumour is exophytic).
- In case of endophytic masses the borders of the tumour are identified with US intra-operative guidance after defatting.
- Complete exposition and defatting of kidney surface except for fat overlying the tumour. Reason: 1. Oncological reason; 2. It will help to exert traction during enucleation / resection.
- Mark the resection line all around the tumour edge according to the endoscopic / intraoperative US appearance of the tumour. This will help later on to find secure landmarks during the excision (also in case of bleeding).
- Mobilize the kidney as much as needed: in this situation, there is the possibility to use some gauze to move gently, tilt and to lift the kidney; the mobilization of the kidney is not only necessary for having the kidney in a favourable position during resection and renorrhaphy, but also for a good closure of the defect, as too much tension will give risk of tearing the parenchyma during suturing.
- **TIME OUT & Clamping the artery (and/or vein) - check if everything is ready for clamping:**
- bulldog clamps or rummel tourniquet
- needle drivers
- sutures (type and length, ready prepared)
- Endoclips (hem-o-locks or similars)
- communicate with the anaesthesiologist for the ischemia time, start and stop to be documented.

**2. Tumour excision (enucleation, enucleo-resection, resection)**

- With non-dominant hand gently lift the perirenal fat overlying the tumor and with dominant hand make a sharp incision on the renal capsule 2/3 mm away from the border from border of the tumour.
- Widen the first sharp incision up to overall 5mm, to allow an easier identification of the tissues. Search for: colour difference compared to the surrounding kidney parenchyma (whitish/yellowish), flat perpendicular surface, cleavable plane. Pay attention if complex cysts to avoid any type of traction and generally should be preferred an enucleo-resection in these cases.
- In case of big masses clamping can be applied after having marked the resection line
- In case of small masses, clamping will be evaluated during enucleation according to surgeon’s experience and to his ability to control bleeding adequately, thus providing a clean working area.
- Produce counter-traction between tumour (non-dominant hand) and parenchyma (dominant hand) to identify and follow the correct cleavage plane and to avoid inadvertent rupture of tumour pseudo capsule.
- In case of enucleation the surgeon will provide a dissection as close as possible to the tumour pseudocapsule by blunt and sharp technique^4^. The latter is preferable whenever the pseudocapsule loses integrity due to adhesions or transcapsular vessels.
- Preserve steady counter-traction of the tumour while deepening the dissection of the enucleation plane. With counter traction, the appropriate plane between the pseudocapsule and the renal parenchyma or the pelvicalyceal system is easily identified.
- Inadvertent cut or entry into the parenchyma or the pelvicalyceal system will be recognized by the loss of the colour continuity of the cutting surface or the increase in bleeding, or the effluence of urine in the operating field, respectively. Inadvertent cut or entry into the tumour should also be recognized by the effluence of necrotic/oncotic material, and/or the more yellowish tissue emerging into the dissecting plane. In order to correctly readjust the plane between the renal parenchyma and the tumour, the surgeon usually steps back (with the camera and the instruments) a few millimeters from the current plane, identifies the normal parenchyma and either dissects superiorly (inadvertent entry into the parenchyma / PC system) or inferiorly (inadvertent entry into the tumour) to the wrong plane. Having difficulties to re-identify or re-routing the correct plane necessitates the creation of a new plane a few centimetres away from the original plane.
- Throughout the procedure the assistant will provide a clean field by rinsing/flushing saline when necessary and/or by pressing down any bleeding points
- Visible bleeding vessels and incidental opening of the collecting system are ligated with running capsular suture with polyglactin, 2/0 thread on a CT-2, 26, 1/2C, taper-point needle.
- An early arterial unclamping could be attempted at this time in order to decrease warm ischemia time. This will eventually help also to identify and fix bleeding points.

1. **Renorrhaphy**

It is highly recommended to pre-plan the suturing based on tumour size, depth and location. The sutures must be pre-fixed and set on the back-table or inserted inside the body cavity and fixed on the posterior abdominal wall before tumour excision (see TIME OUT for clamping).

There are several ways to close the defect:

- One layer in cortical small defects
- Two layers in deeper defects (inner renorrhaphy for vessels)
- Three layers in which the collecting system is closed separately.

**3a Internal renorrhaphy**

- *Two possibilities to proceed after excision of the tumour*
- *Knotless running sliding-clip suturing technique*
- *Knotless interrupted mattress sliding-clip technique*
- *Knotless running sliding-clip suturing technique*

1. A CT-1 ½ circle needle polyglactin/PDS suture is prepared on the back table by applying a hem-o-lok (exactly at its centre) clip to the suture's free end. A prefixed knot has been performed at the free end of the suture and the clip is applied just in front of the knot.
2. The suture must be fixed exactly at the centre of the clip and perpendicularly, this area exerts more firmly and distributes the force.
3. The recommended length of the suture is 18-20cm long, depending on the size of the defect.
4. Based on individual anatomy two such sutures should be pre-fixed and ready for use
5. The suture is then passed through the renal capsule at the edge of the nephrothomy, from the renal parenchyma outside, to the renal “bed” inside. The renal bed is sutured over and over to seal any bleeding vessels or any openings of the pelvicalyceal system. The suture is finally pulled out from the renal bed inside, to the renal parenchyma outside at the opposite edge of the nephrothomy.
6. The suture line is locked by the appliance of a non-absorbable (at the middle of the clip) at the exterior part of the suture in contact with the surface of the renal parenchyma.
7. Gentle traction of the tail of the suture at this point is necessary to avoid loosening of the internal renorrhaphy line.
8. (*At the end of the external renorrhaphy) As the renal parenchyma is re-approximated, the internal renorrhaphy may loosen. At this point, traction on both tails (one at a time) of the internal renorrhaphy line will expose the knot of the suture and the clip applied on each site. Another non-absorbable clip is then applied underneath them (on its corner, we call it the ‘locking’ non-absorbable clip)), in close proximity to the renal capsule in order to reinforce the internal renorrhaphy line.

- *Knotless interrupted mattress sliding-clip technique*

1. Multiple CT-1 ½ circle needle polyglactin/PDS sutures are prepared on the back table by applying a hem-o-lok (exactly at its center) clip to the suture's free end. A prefixed knot has been performed at the free end of the suture and the clip is applied just in front of the knot.
2. The suture must be fixed exactly at the center of the clip, this area exerts more firmly, and perpendicularly.
3. The recommended length of the suture is 10-12cm long.
4. Based on individual anatomy and length of the enucleation bed several such sutures should be pre-fixed and ready for use
5. The first suture is then passed through the renal capsule at the edge of the nephrothomy, from the renal parenchyma outside, to the renal “bed” inside. Next, a separate bite of the renal bed is taken, to seal any bleeding vessels or any openings of the pelvicalyceal system, and the suture is passed from inside out to the opposite site of the renal parenchyma. In such way a mattress suture has been performed. The thread of the suture is locked with a new hem-o-lock clip but the suture is not tightened at this moment but left loose instead. Several such sutures are placed alongside the surface area needed to be re-approximated and remain loose.
6. When all the sutures have been placed, start tighten them one by one by pulling the suture and pressing the hem-o-lock clip at the thread of the suture but also at the tail of the suture against the renal parenchyma so as to reapproximate the inner parenchyma and “lock” the sutures. Repeat the same procedure for all the sutures.

**3b External renorrhaphy**

- Three possibilities to proceed after excision of the tumor
  - Sliding-clips renorrhaphy (running or single sutures)^5,6^
  - Sliding-clips renorrhaphy with barbed suture^7,8^
  - Suture-less, coagulation and biological hemostatic agents without reconstructing the renal parenchyma^9^.
- *Sliding clip technique (running suture):*

1. A CT-1 ½ circle needle polyglactin suture is prepared on the back-table by applying a hem-o-lok exactly at its center) clip to the suture's free end. A prefixed knot has been performed at the free end of the suture and the clip is applied just in front of the knot.
2. The suture must be fixed exactly at the center of the clip, this area exerts more firmly, and perpendicularly.
3. The recommended length of the suture is 18-20cm long.
4. The suture is then passed through the renal capsule perpendicularly and pulled to the desired tension.
5. A second hem-o-lok clip secures it snugly against the opposing renal capsule with the aid of a right-angle forceps.
6. In preparation for the next throw, a new Hem-o-lok clip is applied 1.5 cm proximal to the second set of clips.

- *Knotless suturing with anchoring clips Sliding clip technique (separated sutures):*

1. A CT-1 ½ circle needle polyglactin suture is prepared on the back-table by applying a hem-o-lok exactly at its center) clip to the suture's free end. A prefixed knot has been performed at the free end of the suture and the clip is applied just in front of the knot.
2. The suture must be fixed exactly at the center of the clip, this area exerts more firmly, and perpendicularly.
3. The recommended length of the suture is 12-15cm long.
4. The suture is then passed through the renal capsule perpendicularly and pulled to the desired tension.
5. A second hem-o-lok clip secures it snugly against the opposing renal capsule with the aid of a right-angle forceps.
6. The suture is then cut, the needle is removed and another separate pre-fixed suture is passed through the renal capsule in a 3-5 mm width distance from the entrance of the previous suture.

- Sliding-clips renorrhaphy with barbed suture

Same steps as with the PDS suture

**optional:**

**E. “Do’s and Don’ts”**

- Surgeon:
  - **Do’s:**
    - A proper preoperative planning and adequate pre-operative imaging is critical to define anomalous anatomy and vasculature 3D arterial reconstruction is suggested
    - Get ready to clamp artery whenever attempting zero ischemia technique
    - Rotate the kidney, if necessary, in order to achieve the best geometrical approach to the tumor (remember triangularization of instruments or trying to achieve the best angle to obtain the best surgical technical results).
    - Search for the correct landmarks and find your plane to the preferred excision technique
    - To speed up warm ischemia time
      - Pre-insert sutures before ischemia time
      - Tap the needle tip on the posterior abdominal wall in a way that will leave the proximal one third of the needle free to grasp it with the needle holder of the dominant hand. As such, you will be ready to pass the suture through the renal parenchyma
      - Alternatively, grasp initially the distal one third of the needle with the needle holder of the non-dominant hand. Grasp the needle in a fashion parallel to its curve and expose it to the needle holder of the dominant hand. Most of the time, with a slight rotation of the non-dominant hand, the needle holder of the dominant hand will grasp the needle in a perpendicular way.
      - Alternatively, leave the needle onto the tissue with its “smile” facing upwards and its tip “looking” leftwards. Then grasp it with the needle holder of the dominant hand. Most needle holders will automatically fix the needle in a perpendicular way
      - Always, grasp the needle at the proximal one third and in a perpendicular fashion
      - When the tip of the needle is coming out from the opposite site of the renal parenchyma, grasp it with the non-dominant hand, rotate your wrist to take the needle out form the parenchyma, rotate back your wrist and grasp the needle with the needle holder of the dominant hand. In such way, you do not loose time to readjust the needle.
      - When applying separate sutures you can leave the needles attached without cutting the sutures. You can cut all of them at the end of suturing
      - Alternatively, you can remove the needle while at the same time your assistant is bringing inside the next needle
      - Train yourself in laparoscopic suturing in dry and wet lab prior to real life surgery
      - Ask your assistant to suck properly the renal bed so as to plan your suture line through the bleeding points or the collecting system openings.
    - Remove needles from trocars slowly and under vision. Grasp the suture near the edge of the needle with your dominant hand. Always use a firm needle-holder or a locking grasper to remove the needle.
    - Perform a wise bleeding control after de-clamping. Bring eventually the pneumoperitoneum to 5mm Hg to double check, before closing.
- **Dont’s**
  - Avoid conventional suturing, it provides tangential compression at the capsular edge. The traditional knot tying can also lead to “chees cutting” effect, rendering less compressive force.
  - Approach excision with no prior hilum dissection and arterial isolation.
- Assistant
  - **Assistant:**
- **Do’s**
  - Provide stable camera handling, trying to center the target, in a proper distance
  - Suction is mandatory and important during tumor excision and suturing.
  - Slight downward traction of the renal parenchyma with the suction tip (counter-traction) enables the surgeon to better expose the tumor border.
  - While cutting the tumor, pressing a bleeding point with the suction saves time and reduces blood loss.
  - Aspirate by pressing slightly the suction at the renal bed and by moving back wards the suction on the renal bed
- **Don'ts**
  - Avoid over-aspiration (it decreases pneumoperitoneum and increases venous bleeding).  Preferring rinsing or flushing.
  - During the running suture, be ready with the hem-o-lock clip, insert it promptly, and clip its middle onto the suture.
  - Do not make unnecessary movements during tumor excision or suturing

**F. How to handle problems**

1. **Tumor exposition**

Difficult tumor identification:

- Complete kidney defatting and kidney mobilization
- Ask for the laparoscopic intraoperative ultrasound
- Check again radiologic image: try to find some landmark (i.e. simple cysts)
  1. **Toxic fat: difficult exposition of renal surface**
     - Take your time
     - Blunt dissection with suction

1. **Tumor excision (enucleation, enucleo-resection, resection)**
   1. **Cutting into the tumor:**

- Try to have a clean field
- Switch to warm ischemia from zero ischemia
- Ask anesthesiologist to decrease patient’s blood pressure
- Try to remove it by deepening the resection
  1. **Excessive bleeding from bed of resection**
- Apply gauze temporarily and press slightly
- Ask for raising the pneumoperitoneum to 20

1. **Renorrhaphy**
2. **Cutting cheese effect**

- Avoid excessive traction
- Proceed with the suture

1. **Difficult angle of suture**

- Move the kidney towards you
- Place gauzes in order to stabilize the kidney in favor of your instruments

**TASK idea:**

Model: little box with blood inlet, filled with silicon sponge and a tumor (2 cm). The inlet has a clamp-able tube.

Description: Once the model is prepped with blood bag attached, the trainee decides whether to clamp or not. Time count starts when he/she performs the first cut (he/she might mark the resection line before time starts). He / She performs the whole enucleation. He / She decides how to perform renorrhaphy (internal and/or external). Once he is satisfied with the renorrhaphy, he de-clamps or communicates the tutor that his work is done (in case of no clamp technique)

Target time: clamp (15min) – no clamp (more than “clamp” allowed) (30min)

Blood loss allowed: 250cc (preliminary cut-off to be tested)

Positive margins allowed: none

Time start/stop: first cut/ de-clamp or completion of the renorrhaphy (no clamp technique)

Errors: over time, exceeded blood loss limit, positive margins

**Possible case scenarios:**

- They might do a rough resection to go faster (to be considered as a quality checklist item – appropriate or not)

- They ask to lower blood pressure: tutor partially closes the blood inlet, but reopens fully after the end of the task to check for renorrhaphy tightness.

- They ask for clips: the tutor puts them where they ask for

**REFERENCES**

1. Ljungberg B, Bensalah K, Canfield S, Dabestani S, Hofmann F, Hora M, Kuczyk MA, Lam T, Marconi L, Merseburger AS, Mulders P, Powles T, Staehler M, Volpe A, Bex A.  EAU guidelines on renal cell carcinoma: 2014 update. Eur Urol. 2015 May;67(5):913-24. doi: 10.1016/j.eururo.2015.01.005. Epub 2015 Jan 21. Review.
2. Ljungberg B, Albiges L, Abu-Ghanem Y, Bedke J, Capitanio U, Dabestani S, Fernández-Pello S, Giles RH, Hofmann F, Hora M, Klatte T, Kuusk T, Lam TB, Marconi L, Powles T, Tahbaz R, Volpe A, Bex A. European Association of Urology Guidelines on Renal Cell Carcinoma: The 2022 Update. Eur Urol. 2022 Oct;82(4):399-410. doi: 10.1016/j.eururo.2022.03.006. Epub 2022 Mar 26.
3. B. Ljungberg, L. Albiges, J. Bedke, A. Bex, U. Capitanio, R.H. Giles, M. Hora, T. Klatte T. Lam, L. Marconi, T. Powles, A. Volpe, Y. Abu-Ghanem, S. Dabestani, S. Fernández-Pello Montes, F. Hofmann, T. Kuusk, R. Tahbaz. EAU guidelines on renal cell carcinoma: 2022.
4. Mir MC1, Derweesh I2, Porpiglia F3, Zargar H4, Mottrie A5, Autorino R6. Partial Nephrectomy Versus Radical Nephrectomy for Clinical T1b and T2 Renal Tumors: A Systematic Review and Meta-analysis of Comparative Studies.  Eur Urol. 2017 Apr;71(4):606-617. doi: 10.1016/j.eururo.2016.08.060. Epub 2016 Sep 7.
5. Baumert H, Ballaro A, Shah N, Mansouri D, Zafar N, Molinié V, Neal D. Reducing warm ischaemia time during laparoscopic partial nephrectomy: a prospective comparison of two renal closure techniques. Eur Urol. 2007 Oct;52(4):1164-9. Epub 2007 Mar 28.
6. Häcker A1, Albadour A, Jauker W, Ziegerhofer J, Albquami N, Jeschke S, Leeb K, Janetschek G. Nephron-sparing surgery for renal tumours: acceleration and facilitation of the laparoscopic technique. Eur Urol. 2007 Feb;51(2):358-65. Epub 2006 Aug 7.
7. Bhayani, S.B. & Figenshau, R.S. The Washington University Renorrhaphy for robotic partial nephrectomy: a detailed description of the technique displayed at the 2008 World Robotic Urologic Symposium J Robotic Surg (2008) 2: 139. doi:10.1007/s11701-008-0096-4
8. Shikanov S1, Wille M, Large M, Lifshitz DA, Zorn KC, Shalhav AL, Eggener SE. Knotless closure of the collecting system and renal parenchyma with a novel barbed suture during laparoscopic porcine partial nephrectomy.  J Endourol. 2009 Jul;23(7):1157-60. doi: 10.1089/end.2009.0003.
9. Rassweiler JJ1, Klein J1, Tschada A1, Gözen AS 1  Laparoscopic retroperitoneal partial nephrectomy using an ergonomic chair: demonstration of technique and matched-pair analysis. .BJU Int.  2017 Feb;119(2):349-357. doi: 10.1111/bju.13627. Epub 2016 Sep 12
10. Simone G1, Papalia R, Guaglianone S, Gallucci M. 'Zero ischaemia', sutureless laparoscopic partial nephrectomy for renal tumours with a low nephrometry score.  BJU Int. 2012 Jul;110(1):124-30. doi: 10.1111/j.1464-410X.2011.10782.x. Epub 2011 Dec 16.

# CTA Laparoscopic MVI repair

**A. Indications**

- Vascular injury during dissection is still the most frequent cause of urgent open conversion (1).

**B. Contraindications**

- Low experience of the surgeon (2).
- High blood loss (2).
- Difficult exposure of the area (2).

**C. Equipment (2-7)**

- Absolutely required equipment:
- One 5mm trocar
- Two 10 mm trocars
- One aspiration-irrigation device
- Two 5 mm atraumatic grasping forceps
- One laparoscopic monopolar scissors
- One laparoscopic bipolar forceps
- Laparoscopic Satinsky clamp
- Laparoscopic Crawford clamp
- Laparoscopic clips with applier
- Hem-o-lock clips (M-L, L, XL)
- 3-0 to 5-0 Prolene sutures
- Pre-Prepared sutures with knot at the tail and Hem-o-lock/ Lapra-Ty clip
- Closed suction drain
- 2 Needle holders

Optional equipment:

- Bipolar energy devices for hemostasis and incision (LigaSure, Covidien; EnSeal PTC, Ethicon; Cayman Aescupal, BBrown; etc)
- Ultrasound energy devices for hemostasis and incision (Harmonic scalpel, Ethicon; Thunderbeat, Olympus)

**D. Procedure steps**

- One always has to be prepared for complications, in laparoscopic renal surgery the most significant complications are vascular and bowel-related.
- Early on, while experiencing a major vein injury, decide quickly whether ligation or division of the vein is necessary. For arteries, try to estimate the injury to the circumference.
- *Handling venous injuries*
- Control the bleeding point: compress with a gauze or clamp with an atraumatic grasper and increase the pneumoperitoneum pressure to gain more time to think and control the situation.
- Avoid further damage: remove scissors and avoid applying clips without proper exposure to the bleeding vessel.
- Communicate with your assistant: avoid having the optic lens dirty with blood and avoid damaging the vessel further by applying too much pressure with the suction device.
- Communicate with the anesthesiologists: inform them that there is a major/minor bleeding and, compensate for intravascular volume loss.
- Communicate with the scrub nurse: open try ready, extra trocars, needle drivers, pre-planned prolene sutures.
- Plan your next steps beforehand and make sure the entire team understands and will follow.
- Increase the gas flow to a high level (40 liters/minute) to maintain adequate intraperitoneal pressure (especially in the presence of aggressive suctioning).
- Identification of the injury and further exposure of surrounding structures to allow complete visualization of the region.
- For small tears in the vena cava, pressure plus local hemostatic agents application should be sufficient.
- After adequate exposure has been obtained, in case of a small venotomy, perform a figure-8 stitch, with clips on one side or knot.
- In case of a large injury, consider a temporal clamp of the vessel proximal and distal to the injury. Useful instruments are Satinsky clamp (12mm trocar), curved Crawford clamp (10mm trocar), and Bulldog clams (10-12mm trocars). At this moment consider conversion to open surgery if you found a large defect hard to manage by laparoscopic surgery.
- Repair the injury with interrupted 5-0 Prolene suture (with or without the aforementioned free-knot technique).
- Ask the anaesthesiologist to temporarily lower the blood pressure to 60/70 mmHg (minimal pressure to guarantee regular renal and cerebral perfusion) to allow easier control of the injury.
- *Repairing major arterial injury.*
- Arterial injuries need suturing, grafting, end-to-end anastomosis or bypassing depending on the extent of the injury.
- Arterial lacerations need suturing with 5-0 Prolene suture (simple or figure-8 stitch).
- Injuries that encompass >30% of the circumference of the artery need repair with vein or Gore-Tex patch graphs. Ask for vascular surgery assistance.
- Complete arterial transection requires end-to-end anastomosis.
- More complicated injuries require bypassing vascular surgery.
- Steps 2,3 and 4 require mobilization of the artery and clamping above and below the level of the injury (either with laparoscopic bulldog clamps or with vessel loop tourniquet; see the renal hilum dissection chapter).
- Remember to flush the vessel with heparin before closing the defect.
- In any of the aforementioned steps, vascular surgeon consultation may be needed.

**E. Urgent conversion to open surgery**

In case of (laparoscopic) uncontrollable bleeding, a prompt decision to convert to open surgery should be made. Iodine and sterile cover should already be anticipated on a possible conversion. The patient should be preferably moved to a supine position and a midline or subcostal (Chevron) incision should be made for adequate exposure of major vessels depending on the surgeon's preference.

**F. “Do’s and Don’ts”**

**Do’s:**

- Keep calm
- Take 10 seconds in order to save 10 minutes (debriefing with the team).
- Inform the anaesthesiologist and ask for a close check of patient parameters.
- Keep all instruments in the peritoneal cavity on sight, to avoid a second injury.
- Try to have prompt control of the bleeding vessel and exposure.
- Always have the laparoscopic suture instruments and the laparoscopic applicators available in the room, not necessarily open.
- Remember that using Lapra-Ty or Hem-o-lock clips you can get a 10-12cm length of suture and put the clip on the end of it and you can rapidly sew and follow yourself.
- Maintain a clear view throughout surgery by immediate control of even the slightest bleeding. A bloody surgical field is a dark one with poor vision that promotes errors.
- To prevent a major bleed, you must keep a wide surgical field, with careful dissection and optimal exposure. It is important to optimize your exposure even if that means adding an additional port for retraction and suction.
- Use devices that keep the intra-abdominal pressure steady throughout the operation.

**Don’ts**

- Do not panic. It is not “your blood”. The patient needs you most in case of serious bleeding
- Do not “over-suck” with the suction device. You may lose the intraabdominal pressure and bleed more.
- Don’t place the stitches too far away one from other, to avoid tearing the vessel while suturing.
- Don’t be afraid to convert to open surgery. Be always ready to convert, especially if the bleeding is too brisk or very difficult to manage intracorporeally. Judgement is key; you need to give yourself a little time to realise if the repair will be feasible.

**G. How to handle problems:**

**TASK idea:**

Model: an 8cm-long replica of a vein with a diameter of 2 cm. Materials used need to allow proper stitching without tearing (under average tension). The model has to be perfused from a 3LT synthetic blood bag, with a pressure of 10mmH2O. Before the perfusion is started, the model has to be prepared by the tutor who will perform a vertical +/- 4mm cut at the middle of its length. In case of an examination, the tutor will also double-check the length of the cut in front of the examinee before placing the model inside the box. The tutor checks that the model is properly fixed inside the box.

Description: The trainee gets ready with different instruments available, suggestions are: either two needle holders or one needle holder and a Maryland or Johan graspers. He is provided with a 4-0 prolene, small-needle suture located outside of the laparoscopy box at the start of the exercise. He will be allowed to choose the length of the suture, but the tutor, if asked, will suggest between 10 and 15cm.

When the trainee confirms to be ready, the tutor will fully open the perfusion bag to let the vessel “bleed” and time will start counting. The trainee will start getting the suture inside the laparoscopy box and suturing the injury after the fluid is clearly visible outside of the vessel. The task is considered successfully completed when the needle is out of the laparoscopy training box, and the synthetic vessel starts to expand as the gap is properly sealed (no drops coming out of the injury). The allowed blood loss limit on this exercise will be 1.5 LT.

**Target time:**

Time start / stop: Fluid comes out of the vessel injury / suture cut and needle out of the laparoscopy training box after successfully suturing the defect. Successful suture: the synthetic vessel starts to expand as the gap is properly sealed (no drops coming out of the injury).

**Errors:**

- Blood loss > 1.5 LT.

- Rough handling of the vessel

- Additional damage to the vessel

**Possible case scenarios:**

- Blood pressure looks weak: double check the tubing and the tube locking devices

- The injury produced by the tutor is larger than 4mm: ask for a new model to be prepared

- The model is not correctly fixed to the tray: ask for a check or, in case, for a new model

**REFERENCES**

1) Siqueira TM, Kuo RL, Gardner TA, et al. Major complications in 213 laparoscopic nephrectomy  cases: the Indianapolis experience. J Urol 2002; 168:1361)

2)Fahlenkamp D, Rassweiler J, Fornara P, Frede T, Loening SA. Complications of laparoscopic procedures in urology: experience with 2,407 procedures at 4 German centers. J Urol 1999; 162:765.;

3) Simon S, Castle E, Ferrigni R, et al. Complications of laparoscopic nephrectomy: the Mayo clinic  experience. J Urol 2004; 171:1447

4) Vilos GA. Litigation of laparoscopic major vascular injuries in Canada. J Am Assoc Gynecol  Laparosc 2000; 7:503;

5) Gill IS, Kavoussi LR, et al. Complications of laparoscopic nephrectomy in 185 patients: a multi-  institutional review. J Urol 1995; 154:479;

6)Thiel R, Adams JB, Schulam PG, Moore RG, Kavoussi LR. Venous dissection injuries during laparoscopic urological surgery. J Urol 1996; 155:1874;

7) Rassweiler J, Fornara P, Weber M, et al. Laparoscopic nephrectomy: the experience of the laparoscopy working group of the German Urologic Association. J Urol 1998; 160:18)

# CTA Laparoscopic Vesicourethral anastomosis

1. **Indications:**

Vesicourethral anastomosis (VUA) represents the reconstructive phase of radical prostatectomy. Although final functional outcomes in terms of continence will highly depend on patients’ characteristics, minutely precise dissection and careful surgical plane work, VUA remains a rather delicate and crucial step in radical prostatectomy.

1. **Contraindications:**

Limited experience of the surgical team

Contraindications to radical prostatectomy:

· No indication for curative therapy (life expectancy < 10 years, local or nodal bulky disease, metastatic prostate cancer)

1. **Equipment**

- 1 x monopolar scissor
- 1 x Maryland dissecting forceps
- 2 x Needle holders
- 18-20F silastic catheter
- Sutures: tie together the two ends of twin 3-0 poliglecaprone-25 or polyglactin sutures 15-20cm (alternatively: 2 uni- or a bidirectional barbed suture: V-Loc™, Quill™, Stratafix™). CT-1 needle monofilament suture.

You can use the same sutures on a 5/8 URS needle when available for laparoscopic approach

1. **Procedure steps**

**Laparoscopic vesicourethral anastomosis**

*a.* *Single-knot running vesicourethral technique (van Velthoven technique^2^)*

· **The length of the suture** will depend on the wideness of the size of the bladder neck and might range from 12 cm to 20 cm. If the bladder neck is wide you can reconstruct it by closing the two corners with a figure of 8 suture to create a fish mouth, or with an anterior or posterior bladder closure.

· **The running suture is prepared** on the back table by tying together the two ends of twin dyed sutures of 3-0 poliglecaprone-25 or polyglactin or one bidirectional barbed suture (or two separated barbed sutures without tying them at the end; running sutures but alternative to Van Velthoven technique)

· **Step 1**: Place both needles outside-in through the bladder neck and inside-out on the urethra, the right needle at 5:30-o’clock and the left needle at 6:30-o’clock position

· **Step 2:** Run the left suture from 6:30 toward 9:00-o’clock position

· **Step 3:** Run the right suture from 5:30 toward 3:00-o’clock position

The last bite-turn of the suture at this moment should come out from the urethra

· **Step 4:** The sutures are then cinched down with gentle traction on each thread either simultaneously or alternatively in order to bring the bladder neck adjacent to the urethra without leaving a gap within the dorsal part of the anastomosis. Apply gentle traction of each thread alternately until a no gap posterior wall is formed

Avoid tearing the urethra by having the suture pass between the two jaws of the open needle holder placed adjacent to the urethra.

· **Step 5:** Place a 18-20F Foley catheter into the bladder

· **Step 6:** Sutures are then passed again outside-in on the bladder neck and inside-out on the urethra running from the 06:30 and 5:30-o’clock positions towards the 10:00 and 2:00 positions (pay attention to avoid catching ureters in bladder neck and check the integrity of ureteral orifice before start)

· **Step 7 (a or b)**

**Option a:** Pass the suture inside-out and then outside-in on the urethra at 2:00 and 10:00- o’clock position and then inside-out the bladder neck in order to perform a U-turn. It allows the stitch to be placed on more resistant bladder outer surface (This step is optional)

**Option b:** Sutures are then continued to the 12:00-o'clock position and tied to each other so that the knot rests on the exterior of the bladder. Barbed sutures do not require a knot to lock.

· Perform an anterior tennis-racket suture if there is a mismatch between bladder and urethra at this stage.

· A new 18 Fr catheter is now placed and the anastomosis.

· **Step 8:** The balloon is inflated and the integrity of the anastomosis is tested by filling the bladder with 150 ml saline.

· Note: the first sutures might be also applied starting from the 4:30 o’clock position for right handed or 7:30 position for left handed, in order to perform the posterior wall suture with the dominant hand.

*b.* *Single running suture*

A single running technique as an alternative to the ‘van Velthhoven technique’ has been described as a simplified technique for facilitating vesicourethral anastomosis. *In order for the reader not to be confused he should consider the hours of the bladder neck like the hours of the open chain clock* (so, in this technique the 12^th^ hour of the bladder neck is the traditional 6^th^ hour)^3^.

- A 15-cm-long absorbable suture (2-0 or 3-0) poliglecaprone with a 3/8 arc needle is prepared on the back table
- The first suture is passed by passing the needle from the outside-in on the bladder neck at the 9-o’clock position and from the inside out on the full thickness of the urethra at the 9-o’clock position
- The suture is then tied into a knot with the suture tail
- Following the first step, the suture is then passed from the outside-in on the bladder neck at the 11 ο'-o'clock position and from inside-out on the urethra at the 7 o’clock position
- The single suture is then continuously passed on the bladder neck at the 1 o’clock position and on the urethra at the 5 o’clock position and on the urethra at the 1 o’clock position an on the bladder neck at the 7 o’clock position and on the urethra at the 11 o ‘clock position
- A new 20 Fr catheter is placed into the bladder and a knot is tied with the suture tail at the 9 o’clock position
- The balloon is inflated and the integrity of the anastomosis is tested by filling the bladder with 150 ml saline

*c.* *Interrupted suture*

The interrupted technique was the first technique that was used for the anastomosis and was initially described by the group of Guillonneau and Vallacien from the Montsouri Institute^4^

- The anastomosis is performed with a 3-0 resorbable 4/8 or 5/8 suture on a No.26 needle
- In the initial original description of the technique, the authors stated that the knots could formed either inside or outside of the anastomotic lumen and a metal Benique catheter guide was used to allow the needle to slide
- The first suture is placed inside-out on the urethra and outside- in on the bladder neck at the 5 o’clock position.
- The suture is then tied inside the urethral lumen
- The second suture is placed inside-out on the urethra and outside-in on the bladder neck at the 7 o’clock position
- Again, the suture is tied inside the urethral lumen
- Then, 4 sutures are symmetrically placed at the 4,8, 2 and 10 o’clock position and tied outside the lumen
- The final 2 sutures are placed outside-in on the urethra and inside-out on the bladder neck at the 11 and 1 o’clock positions
- These sutures are not tied immediately but only after the insertion of the Foley catheter
- The balloon is then inflated and the integrity of the anastomosis is tested by filling the bladder with 150 ml saline.

The interrupted technique has also been described by Leipzig team in the description of the Endoscopic Extrapertioneal Radical Prostatectomy (EERPE)^5^

- Depending on the size of the bladder neck, eight or nine sutures are necessary for a watertight anastomosis. In the case of a widely open bladder neck, bladder neck closure (ventrally) should also be performed.
- The anastomosis is performed with a 2–0 Polysorb suture on a GU-46 needle (alternative: UR-6 needle, 2–0 Vicryl). The bladder neck is always stitched first. All stitches are performed “outside-in” at the bladder neck and “inside-out” at the urethra. In this way the sutures are always tied extraluminally. The first stitch starts at the 8 o’clock position (backhand–backhand). When starting the anastomosis the Trendelenburg position is reduced to a minimum required.
- Secure the knot of the first suture before full approximation. Make sure that the bladder is empty. The final approximation can be reached with the next stitch.
- The next stitches are positioned at the 7, 6 and 5 o’clock positions (forehand at the bladder neck, backhand at the urethra). When stitching the urethra during the posterior anastomosis the urethral catheter always needs to be lifted up by the assistant or the surgeon to guide suture positioning.
- The 4 o’clock stitch is then performed forehand (bladder-neck) – forehand (urethra). In nerve-sparing procedures take care not to include in the suturing the neurovascular bundles (especially the 8 and 4 o’clock stitches are dangerous). The assistant should guide the stitch with the help of the suction.
- After the dorsal circumference has been completed, the final silicone catheter (18–20 F) is placed into the bladder. This is the test for the quality of the posterior part of the anastomosis. If there is a problem the catheter does not slide into the bladder and finds its way through the stitches behind the bladder you then have to revise the anastomosis.
- The anastomosis is now continued laterally on both sides. On the left side (9 o’clock) the stitches are thrown backhand–backhand and on the right side (3 o’clock) forehand–forehand. These stitches are relatively easy to perform and should be performed in one step (stitch the bladder and urethra in one move).
- If during the bladder neck dissection a bladder neck-preserving technique is not feasible, a bladder neck reconstruction at a 12 o’clock position is deemed necessary at this point. Use a running suture with the same needle and suture material. Alternatively, single stitches can be placed. Make sure that the stitches are full thickness on the bladder wall.
- The final two anastomotic sutures are placed at 11 and 1 o’clock positions (left side: backhand–backhand, right side: forehand–forehand). For the 11 o’clock stitch the needle holder is introduced through the right medial 5-mm trocar (on the assistant’s side). This stitch is thrown backhand at the bladder neck and backhand at the urethra, and can be performed in one or two moves. For knot tying the needle holder is moved back to its ini- tial position.
- When suturing the urethra these stitches (11 o’clock and 1 o’clock) should not include the whole tissue of the urethra. They should embrace the Santorini plexus, connective tissue and puboprostatic ligament (not through the mucosa and the musculature of the urethra), thus avoiding any damage to the external (urethral) sphincter and its blood supply and finally fixing the “new” bladder neck to its anatomical position.
- After conclusion of the stitching process the catheter must be moved to make sure that there is no entrapment within the suture lines (very rare). The water-tightness of the anastomosis is finally checked by filling the bladder with 200 ml sterile water. Lateral and ventral leaks can be managed by additional suturing. In the case of a major posterior leak the anastomosis needs to be opened and performed again.

**Robot-assisted vesicourethral anastomosis**

*a.* *Single-knot running vesicourethral technique (van Velthoven technique)*

As described above. Most common technique.

*b.* *Vattikuti Institute technique*

· Two 3-0 barbed sutures (6-inches, RB-1 needle)

· Similar to single-knot running technique

· Suture starts at 4:00-o’clock position

· Direction of the stitch is altered at 9:00-o’clock position and runs to 11:00-o’clock position

· Sutures find each other at 11:00-o’clock position

· Needles are cut without tying knots

· Urethral catheter is removed and a percutaneous suprapubic tube is placed into the bladder

**Important points for all the different types of anastomosis:**

*Posterior musculofascial reconstruction*

· Preserve the median raphe posterior to the urethra during apex dissection

· Place two polyglactin 3-0 sutures through median raphe

· Posterior median raphe is fixed by running suture to the edge of the Denonvilliers’s fascia and then to the posterior bladder wall

*Periurethral suspension stitch*

· After ligation of DVC, place a CT-1 needle monofilament suture passed from right to left between the urethra and DVC

· Pass the same stitch through the periosteum on the pubic bone

· The suture is passed again through DVC and pubic bone in a figure of O

· Tie the suture without tension

*Tips and tricks for the anastomosis per se*

· It is highly recommended, before starting the anastomosis to thoroughly overlook the bladder neck and localize (if visible) the ureteral orifices

· When suturing the urethra, an assistant should press the perineum of the patient and insert the catheter to facilitate stitching?

· After each urethral stitch, the catheter needs to be pulled back in order to rule out inadvertent fixation by the anastomotic suture.

· In order to optimize knot security and avoid knot loosening and slipping, particularly if a synthetic not barbed suture is used, the assistant might be asked to hold the first knot with an atraumatic laparoscopic grasper and keep it secured until the second knot is settled down and tied

· Alternatively (or additionally) when starting the anastomosis reduce the Trendelenburg position to a minimum required

· During the placement of anastomotic sutures the nurse should be asked to move the tip of urethral Foley in an out of the urethral stump to prevent accidental suturing of the catheter

· The final knot should always be tied before the balloon of the catheter is inflated

· Barbed sutures not need to be tied

1. **Do’s and Don’ts**

**Do’s**

· Bleeding control and exposure areis essential to perform a vesicourethral anastomosis

· Inspect the bladder neck with special attention to ureteral orifices

· Choose between bladder neck preservation or reconstruction accordingly to clinical/pathological features

· Preserve a good urethral stump

· Precise bladder neck – urethral stump alignment

· Perform a full thickness (including bladder mucosa) suture

· Perform a tension-free anastomosis

· Non-ischemic watertight suture

· Easy learning technique

· Perform a full thickness (including bladder mucosa) suture

· Perform posterior musculofascial reconstruction and periurethral suspension stitch aiming higher early urinary continence rates

· Fill the bladder after urinary reconstruction with saline (120-180ml) to test the integrity of the anastomosis

· The use of new technologies as ETHOS surgical chair, three-dimensional laparoscopy and mechanical manipulators (Radius Surgical System) may be helpful to handle laparoscopic vesicourethral anastomosis

**Don’ts**

· Avoid over-aspiration: it reduces pneumoperitoneum and increases venous bleeding; it disturbs the surgeon, pelvis is a narrow space

· Avoid bladder over-traction, especially after posterior wall of anastomosis is completed

· Avoid tension. Release the bladder as much as necessary

· Be careful about clips next to vesicourethral anastomosis to avoid clip migration

· Do not attempt to approximate the bladder to the urethra in a single step.

1. **How to handle problems**

a. Urinary leakage

· Ensure good exposure and bleeding control before performing the anastomosis

· Ensure there is no blood clot inside the bladder. Catheter obstruction can lead to urinary leakage

· Interrupted vs. running or monofilament vs. barbed sutures do not play a role in urinary leakage

· Posterior musculofascial reconstruction reduces anastomotic tension

· Check the integrity of the anastomosis by intraoperative flush test. Additional sutures or even complete revision of the anastomosis can be necessary.

· Checking for a tight posterior wall suture will allow to eventually have leakage just from the anterior side (much easier to repair)

**TASK idea:**

**Model:** A silicone male pelvic model is connected to a lap training box. A urethra is coming from distally, entering the pelvis, where a bladder with bladder neck is mobile in place and ready to be anastomosed. Two orifices can be identified at the trigone, close to the bladder neck. The urethra has to be accessible to place a Foley catheter, the bladder needs to be a closed area so that water tightness of the anastomosis can be checked after suturing.

**Description:** The trainee gets ready with two needle drivers and a suture. The trainee can choose one of the sutures as described above. The time starts when the trainee enters the pelvis with the suture. The trainee will perform the suturing and put the catheter in place. When ready the trainee communicates this with the tutor and the time stops. The tutor then will test the anastomosis for quality and for leakage by filling the bladder with water through the Foley catheter.

**Target time:** 20-30 min

**Errors:**

-Loose anastomosis showing leakage after testing

-Knotting inside of the bladder/urethra

-Suturing the orifices

-Open bladder neck and/or urethra

References

1. Albisinni S, Aoun F, Peltier A, Van Velthoven R. The Single-Knot Running Vesicourethral Anastomosis after Minimally Invasive Prostatectomy: Review of the Technique and Its Modifications, Tips, and Pitfalls. Prostate Cancer. 2016;2016. doi:10.1155/2016/1481727
2. Van Velthoven RF, Ahlering TE, Peltier A, Skarecky DW, Clayman RV. Technique for laparoscopic running urethrovesical anastomosis:the single knot method. Urology. 2003 Apr;61(4):699-702. doi: 10.1016/s0090-4295(02)02543-8. PMID: 12670546.
3. Yang J, Shao PF, Lv Q, Song NH, Li J, Zhang W, Li P, Hua LX, Yin CJ. Continuous suture of a single absorbable suture: a new simplified vesicourethral anastomosis technique in laparoscopic radical prostatectomy. Int Surg. 2014 Sep-Oct;99(5):656-61. doi: 10.9738/INTSURG-D-13-00124.1. PMID: 25216438; PMCID: PMC4253941.
4. Guillonneau B, Vallancien G. Laparoscopic radical prostatectomy: the Montsouris technique. J Urol. 2000 Jun;163(6):1643-9. doi:10.1016/s0022-5347(05)67512-x. PMID: 10799152.
5. Stolzenburg JU, Gettman MT, Liatsikos E (2007). Endoscopic Extraperitoneal Radical Prostatectomy: Laparoscopic and Robot-Assisted Surgery. Springer Berlin.
6. Ghani KR, Trinh Q-D, Menon M. Vattikuti Institute Prostatectomy—Technique in 2012. *J Endourol*. 2012;26(12):1558-1565. doi:10.1089/end.2012.0455.
7. Montorsi F, Wilson TG, Rosen RC, et al. Best practices in robot-assisted radical prostatectomy: Recommendations of the Pasadena consensus panel. *Eur Urol*. 2012;62(3):368-381. doi:10.1016/j.eururo.2012.05.057
8. Williams SB, Alemozaffar M, Lei Y, et al. Randomized controlled trial of barbed polyglyconate versus polyglactin suture for robot-assisted laparoscopic prostatectomy anastomosis: Technique and outcomes. *Eur Urol*. 2010;58(6):875-881. doi:10.1016/j.eururo.2010.07.021.
9. Patel VR, Coelho RF, Palmer KJ, Rocco B. Periurethral Suspension Stitch During Robot-Assisted Laparoscopic Radical Prostatectomy: Description of the Technique and Continence Outcomes. *Eur Urol*. 2009;56(3):472-478. doi:10.1016/j.eururo.2009.06.007.
10. Bernardo Rocco, Gabriele Cozzi, Matteo G. Spinelli, Rafael F. Coelho, Vipul R. Patel, Ashutosh Tewari, Peter Wiklundf, Markus Graefen, Alex Mottrie, Franco Gaboardi, Inderbir S. Gill, Francesco Montorsi, Walter Artibani FR. Posterior musculofascial reconstruction after radical prostatectomy: an updated systematic review and a meta-analysis. *Eur Urol*. 2012;62:779-790.
11. Cathelineau X, Sanchez-Salas R, Barret E, et al. Radical prostatectomy: evolution of surgical technique from the laparoscopic point of view. *Int Braz J Urol*. 2010;36(2):129-139. doi:10.1590/S1677-55382010000200002.
12. Cathelineau X, Sanchez-Salas R, Barret E, et al. Radical prostatectomy: evolution of surgical technique from the laparoscopic point of view. *Int Braz J Urol*. 2010;36(2):129-139. doi:10.1590/S1677-55382010000200002.
13. Tyritzis SI, Katafigiotis I, Constantinides CA. All you need to know about urethrovesical anastomotic urinary leakage following radical prostatectomy. *J Urol*. 2012;188(2):369-376. doi:10.1016/j.juro.2012.03.126.
14. Valero R, Schatloff O, Chauhan S, et al. Sutura barbada bidireccional para la reconstrucción del cuello vesical, la reconstrucción posterior y la anastomosis vesicouretral durante la prostatectomía radical asistida por robot. *Actas Urológicas Españolas*. 2012;36(2):69-74. doi:10.1016/j.acuro.2011.06.010.
15. Tokas T, Gözen AS, Avgeris M, et al. Combining of ETHOS Operating Ergonomic Platform, Three-dimensional Laparoscopic Camera, and Radius Surgical System Manipulators Improves Ergonomy in Urologic Laparoscopy: Comparison with Conventional Laparoscopy and da Vinci in a Pelvi Trainer. *Eur Urol Focus*. 2016;4569(16):10-11. doi:10.1016/j.euf.2016.11.006.

# Supplementary Table 1. Delphi Round Participation

| Delphi Topic | Invited | Round 1 Completed Surveys | Round 1 Uncompleted Surveys | Round 2 Completed Surveys | Round 2 Uncompleted Surveys |
| --- | --- | --- | --- | --- | --- |
| Enucleation | 72 | 61 | 1 | 59 | 2 |
| Pyeloplasty | 72 | 60 | 1 | 60 | 1 |
| VU Anastomosis | 73 | 62 | 2 | 59 | 2 |
| Hilum Dissection & MVI | 73 | 64 | 2 | 63 | 2 |
